# Optimized Electronic Modification of S-Doped CuO Induced by Oxidative Reconstruction for Coupling Glycerol Electrooxidation with Hydrogen Evolution

**DOI:** 10.1007/s40820-023-01159-6

**Published:** 2023-07-29

**Authors:** Ruo-Yao Fan, Xue-Jun Zhai, Wei-Zhen Qiao, Yu-Sheng Zhang, Ning Yu, Na Xu, Qian-Xi Lv, Yong-Ming Chai, Bin Dong

**Affiliations:** China State Key Laboratory of Heavy Oil Processing, College of Chemistry and Chemical Engineering, China University of Petroleum (East China), Qingdao, 266580 People’s Republic of China

**Keywords:** Glycerol oxidation reaction (GOR), Hydrogen evolution reaction (HER), CuO, Oxidative reconstruction, Electronic modification

## Abstract

Glycerol (electrochemical) oxidation reaction (GOR) producing organic small molecule acid and coupling with hydrogen evolution reaction is a critical aspect of ensuring balanced glycerol capacity and promoting hydrogen generation on a large scale. However, the development of highly efficient and selective non-noble metal-based GOR electrocatalysts is still a key problem. Here, an S-doped CuO nanorod array catalyst (S-CuO/CF) constructed by sulfur leaching and oxidative remodeling is used to drive GOR at low potentials: It requires potentials of only 1.23 and 1.33 V versus RHE to provide currents of 100 and 500 mA cm^−2^, respectively. Moreover, it shows satisfactory comprehensive performance (at 100 mA cm^−2^, *V*_cell_ = 1.37 V) when assembled as the anode in asymmetric coupled electrolytic cell. Furthermore, we propose a detailed cycle reaction pathway (in alkaline environment) of S-doped CuO surface promoting GOR to produce formic acid and glycolic acid. Among them, the C–C bond breaking and lattice oxygen deintercalation steps frequently involved in the reaction pathway are the key factors to determine the catalytic performance and product selectivity. This research provides valuable guidance for the development of transition metal-based electrocatalysts for GOR and valuable insights into the glycerol oxidation cycle reaction pathway.

**Supplementary Information:**

The online version contains supplementary material available at 10.1007/s40820-023-01159-6.

## Highlights

- S-doped CuO nanorod arrays (S-CuO/CF) constructed by sulfur leaching and oxidative remodeling strategy require only 1.23 and 1.33 V versus hydrogen evolution reaction (HER) to provide glycerol oxidation currents of 100 and 500 mA cm^−2^.
- S-CuO/CF shows satisfactory performance (at 100 mA cm^−2^, *V*_cell_ = 1.37 V) assembled as the anode in asymmetric coupled electrolytic cell of glycerol oxidation reaction and HER.
- The study identifies the key factors involved in the GOR reaction pathway, which include the C–C bond breaking and lattice oxygen deintercalation steps.

**Supplementary Information:**

The online version contains supplementary material available at 10.1007/s40820-023-01159-6.

## Graphical abstract

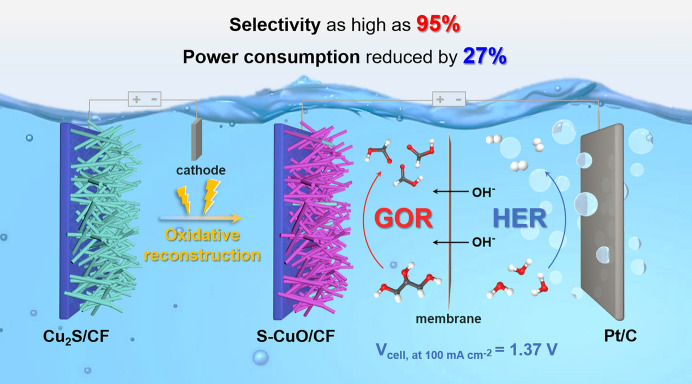

## Introduction

Hydrogen energy is considered an eco-friendly and highly valuable energy carrier that can provide a breakthrough in achieving carbon neutrality, overcoming energy crises, and protecting the environment [1–3]. Compared with hydrogen production via heavy industry, such as fossil fuel hydrogen generation, steam methane reforming and industrial by-product hydrogen extraction, the dazzling advantages of low environmental risks and high hydrogen purity of water electrolysis technology for hydrogen production have aroused high research enthusiasm [4–7]. But at present the key bottleneck of the development of electrocatalytic water splitting technology lies on the huge energy consumption caused by the high-overpotential oxygen evolution reaction (OER) [8–10]. Even if the reaction overpotentials of hydrogen and oxygen evolution are not taken into account, the theoretical decomposition potential of water reaches 1.23 V, which is an absolute low ebb that can never be broken through the improvement of electrolytic water catalysts. In the long run, there is no value-added room for oxygen. And for industrial electrolyzer units, the biggest destroyers of alkaline membrane are the reactive oxygen species produced in the process of OER [11, 12]. Within the compacted apparatus, the potential consequences of membrane damage are significant. Not only is there risk for electrode short-circuiting, but additionally, the mixing of high-density oxygen and hydrogen presents a serious concern [13, 14]. Therefore, it is an effective way to choose some other oxidation reactions of liquid molecules with low oxidized potential coupled with hydrogen evolution reaction (HER) to reduce the overall energy demand and improve the safety factor of hydrogen production. Recently, many studies have reported that some more easily oxidized small molecule compounds such as hydrazine [15–17], urea [18, 19], and furfural [20–22] can be used as sacrificial agents to replace OER at the anode. Although they can effectively save the input potential of the anode, their own cost is still a concern.

Glycerol is an abundant and inexpensive small organic molecule, which can be used as an ideal anode reactant. First of all, as a by-product of the biodiesel industry, the production of glycerol has reached a surplus state [23]. To make the most of cheap glycerol, the U.S. Department of Energy has identified it as a critical platform molecule of conversion into higher-value products [24]. A variety of value-added chemicals such as 2-hydroxyacetone, glyceric acid, glycolic acid, formic acid, etc., can be produced by glycerol oxidation reaction (GOR) [25–27]. In addition, the usual oxidation potential of glycerol is much lower than the theoretical OER potential (1.23 V vs. standard hydrogen electrode (SHE)) under standard conditions. More specifically, the complete oxidation of a glycerol molecule into formic acid requires only a theoretical potential of 0.69 V versus SHE, even though the process involves breaking the C–C bond [28]. Simultaneously, the production of three times formic acid with one equivalent of glycerol at low energy consumption is an attractive way to meet the needs of large-scale formic acid fuel cells.

Recently, scholars have reported some research works on electrooxidation of glycerol. For example, Han et al. prepared a series of nanostructures of cobalt-based spinel oxides (MCo_2_O_4_, M = Mn, Fe, Co, Ni, Cu and Zn), demonstrating that CuCo_2_O_4_ has the highest intrinsic catalytic activity for the selective oxidation of glycerol to formic acid in alkaline environment (pH = 13). And they achieved formic acid production selectivity and conversion of 80.6% and 79.7% [29]. Li et al. reported that Ni–Mo–N/CFC nanosheets were integrated into an alkaline electrolyzer as a bifunction electrocatalyst for glycerol oxidation and hydrogen evolution reactions, which only required 1.36 V potential to provide a current density of 10 mA cm^−2^ [30]. Furthermore, He et al. combined in situ Raman spectroscopy techniques and density functional theory (DFT) calculations to reveal the detailed reaction mechanism of glycerol oxidation catalyzed by transition metal hydroxides [28]. They emphasized that the process of deintercalation of proton and oxygen anion from the catalyst surface will determine the reactivity of the reaction. The surface of Co-doped Ni-hydroxide exhibits a significantly favorable trend of ion deintercalation, thus providing the best formic acid yield. Currently, despite some studies illustrating that certain intricately engineered materials may offer superior glycerol oxidation activity, the actual catalytic reaction mechanism is yet to be fully elucidated. In short, the development and research of transition metal-based electrocatalysts for GOR are still challenging, especially considering the autooxidation and reconfiguration process of transition metals at the anode.

Transition metal-based sulfides inevitably undergo remodeling process during anodic oxidation, and the resulting reconstituted interface shows enhanced catalytic activity in various reactions. Herein, we report an effective strategy for preparing sulfur-doped copper oxide nanorods (S-CuO/CF) on copper foam (CF) substrate by in situ electrooxidation reconstruction of cuprous sulfide precursors (Cu_2_S/CF). Under oxidation current, the surface leaching behavior of sulfur ions induces a rapid remodeling process, and the resulting S-doped CuO has wider active surface, faster interfacial electron transfer and higher GOR catalytic activity. Moreover, S-CuO has a more moderate deintercalation ability of surface oxygen anions, which promotes the directional transition of reaction intermediates to provide higher selectivity. In this work, S-CuO/CF acts as an efficient anodic electrocatalyst to selectively promote the electrooxidation of glycerol to value-added formic acid, requiring a potential of only 1.23 V versus RHE to achieve a current density of 100 mA cm^−2^. Furthermore, when integrated as an anode into an alkaline electrolyzer, it can provide 100 mA cm^−2^ at a relatively low potential of 1.37 V, an improvement of 500 mV over alkaline water splitting. The in situ oxidative remodeling strategy proposed in this work provides a novel design idea and development direction for transition metal-based GOR electrocatalysts to achieve efficient formate production and low power cost hydrogen generation.

## Experimental Section

### Materials

All chemicals, including potassium persulfate [K_2_S_2_O_8_] (≥ 98.5% AR), sodium hydroxide [NaOH] (≥ 96%), acetone [C_3_H_6_O] (≥ 99.5% AR), hydrochloric acid [HCl] (1 M, GR) and ethyl alcohol [C_2_H_6_O] (≥ 99.7% AR) were purchased from Sigma-Aldrich and used as received without any purification. Copper foam (CF) is also purchased from merchants, and its thickness is 1.5 mm with a porosity of 130 pores per linear inch (ppi). Prior to synthesis, the CF was ultrasonic cleaned in acetone, hydrochloric acid and ethanol for 10 min to remove surface impurities and oxides.

### Preparation of Cu(OH)_2_/CF, Cu_2_S/CF and S-CuO/CF

#### Synthesis of Cu(OH)_2_/CF

Cu(OH)_2_/CF was prepared by a simple alkali etching-in situ growth method. A solution containing 2.5 M NaOH and 0.125 M K_2_S_2_O_8_ was prepared and the CF (10 mm × 20 mm × 1.5 mm) was immersed in it for 20 min. Cu(OH)_2_/CF with blue surface was obtained after cleaning with ethanol and deionized water.

#### Synthesis of Cu_2_S/CF

Cu_2_S/CF was prepared by traditional hydrothermal vulcanization method. More specifically, 1 g of Na_2_S·9H_2_O is dissolved in 32 mL of deionized water to form a clear solution. After transferring the above solution to a 100 mL hydrothermal crystallization reactor, a piece of Cu(OH)_2_/CF (2 cm^2^) was immersed and kept at 100 °C for 4 h. After natural cooling, the obtained black Cu_2_S/CF was rinsed with deionized water and dried.

#### Synthesis of S-CuO/CF

S-CuO/CF is obtained by rapid electrochemical activation. The conversion of Cu_2_S to S-CuO (1 cm^2^) was accomplished using linear sweep voltammetry (LSV) within the range of 0–0.85 V versus Hg/HgO potential in 1 M KOH. The electrochemical conditions in question are specified as follows: 5 mV s^−1^, 0–0.85 V versus Hg/HgO, 4 scans, with iR correction (current interrupt (CI) compensation).

## Results and Discussion

### Design Principle and Structural Characterizations

**Fig. 1:**
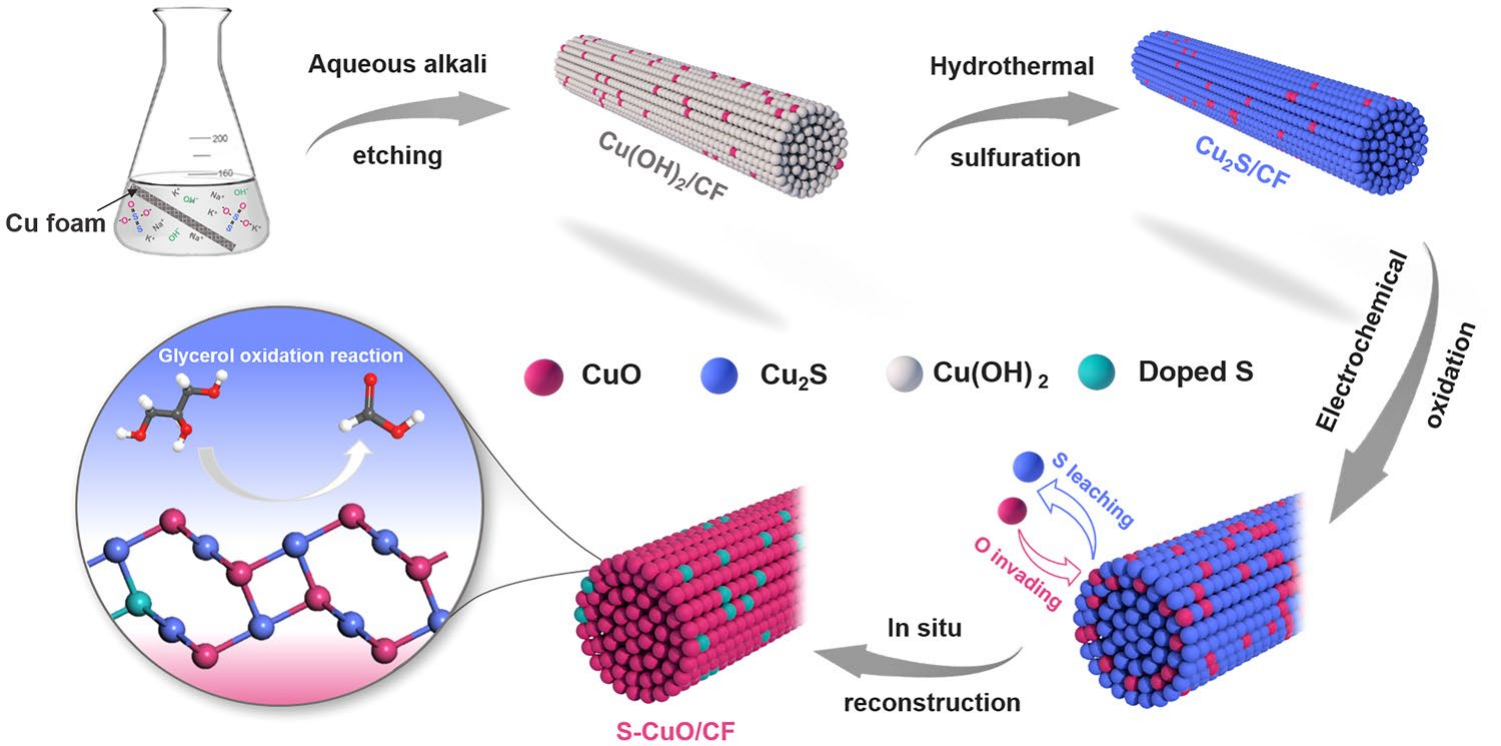
Schematic diagram of preparation of S-doped CuO nanorods by sulfur leaching and oxidation remodeling

**Fig. 2:**
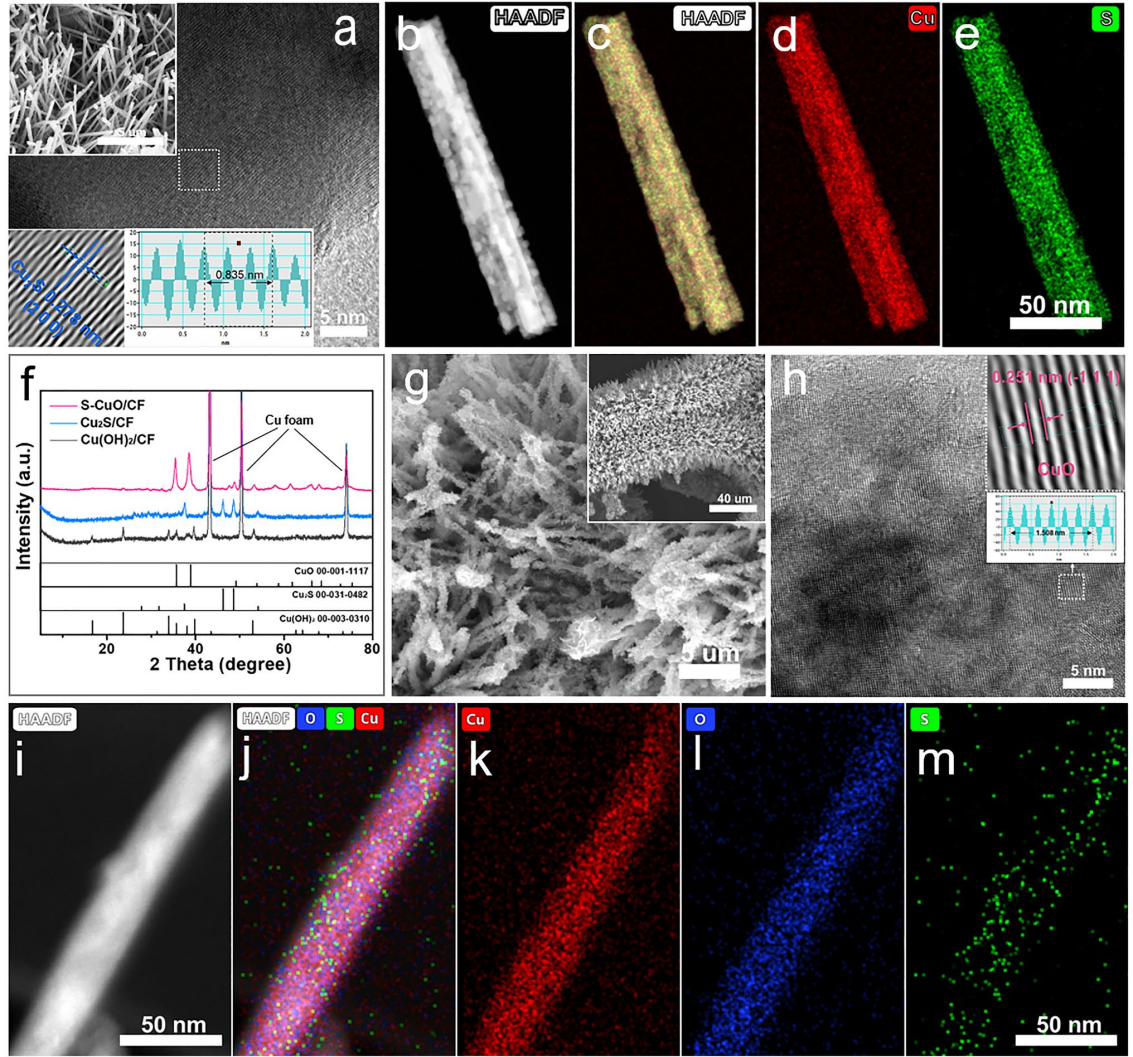
**a** HRTEM (the insets are SEM of Cu_2_S/CF and lattice fringes of Cu_2_S (2 0 0)) and **b**–**e** HAADF-STEM elemental mapping images (Cu and S) of Cu_2_S/CF. **f** XRD pattern of S-CuO/CF, Cu_2_S/CF and Cu(OH)_2_/CF. **g** SEM, **h** TEM (the inset shows lattice fringes of CuO (-1 1 1)), **i** and **j** HAADF-STEM image and **k**–**m** the corresponding EDS elemental mapping for Cu (red), O (blue) and S (green) of S-CuO/CF

The S-CuO/CF nanorods array structure was prepared by self-etching and in situ electrooxidation reconstruction, as shown in Fig. 1. First, a self-etching process occurs on the smooth surface of copper foam (Fig. S1) in alkaline solution (NaOH), and the resulting Cu^2+^ reacts with the abundant OH^−^ in the solution environment to form Cu(OH)_2_ rod-like structures. Figure S2 shows that the surface of CF is tightly covered by a large number of vertically growing Cu(OH)_2_ nanorods to effectively increase its active surface area. Cu(OH)_2_/CF was then reduced to Cu_2_S/CF by a mild hydrothermal vulcanization process, in which the rod-like structure of the precursor was intact (Fig. S3). High resolution transmission electron microscopy (HRTEM) images showed the visible lattice fringes of Cu_2_S (2 0 0) (JCPDS No. 00-031-0482) at a spacing of 0.278 nm (Fig. 2a). In Fig. 2b–e, the corresponding element mapping also show uniform distributions of Cu and S elements. The atomic percentage of O is only 4.33% (Fig. S4), which results from unavoidable surface oxidation, indicating that Cu(OH)_2_/CF has been completely converted to Cu_2_S/CF. Finally, Cu_2_S nanorods can be rapidly transformed into S-doped CuO through electrochemical oxidation and reconstruction process in alkaline environment (1 M KOH). The S leaching and O invading occurred simultaneously during this three linear sweep voltammetry (LSV) cycles. Due to the brief activation period, some internal S cannot be completely leached out and remains in the CuO structure as a doped element. In Fig. 2f, X-ray diffraction (XRD) was used to trace the phase transitions in the above synthesis processes. Among them, Cu(OH)_2_/CF, Cu_2_S/CF and S-CuO/CF are precursors, intermediates and final products respectively, which display similar diffraction patterns matching with Cu(OH)_2_ (JCPDS No. 00-003-0310), Cu_2_S (JCPDS No. 00-031-0482) and CuO (JCPDS No. 00-001-1117), respectively. Within the group of samples, it is observed that the XRD pattern obtained from the S-CuO/CF includes a few minor erratic peaks. These deviations are due to a small number of Cu_2_S remnants not being able to access the electrolyte as a result of the self-supported electrode assembly method. However, they are not expected to have a significant impact on performance. Concurrently, the ultimate sample, as well as other comparable samples were examined by Fourier transform infrared (FTIR) spectroscopy (Fig. S5). In the assessment of the test curve of Cu(OH)_2_/CF, we observed four peaks, ascribed to stretching vibration of structural O–H (O–H_str_, *ν*), stretching vibration of adsorbed O–H (O–H_ads_, *ν*), O–H in-plane bending vibration of adsorbed water (HO–H_ads_, *δ*_in_) and O–H out-of-plane bending vibration of adsorbed water (HO–H_ads_, *δ*_out_), affirming the successful preparation of Cu(OH)_2_ [31, 32]. Cu_2_S/CF does not have significant infrared signature peaks due to the non-infrared active vibrations it provides. In the detection curves of CuO/CF, two peaks of O–H_ads_, *ν* and HO–H_ads_, *δ*_in_ of adsorbed H_2_O appeared, which are derived from water molecules adsorbed on the surface in a humid environment. For S-CuO/CF, the three peaks at 3390, 1639 and 1365 cm^−1^ are attributed to O–H_ads_, *ν*, O–H tensile vibration of adsorbed hydroxyl groups (–O–H_ads_, *ν*) and HO–H_ads_, *δ*_in_. Since more water molecules and hydroxyl groups are adsorbed on the surface of S-CuO/CF after contact with the electrolyte in the process of electrochemical activation, it has stronger O–H_ads_, *ν* vibration peak. Scanning electron microscopy (SEM) images of as-synthesized S-CuO sample showed that the surface of the vertically distributed nanorods became significantly rough after the electrochemical oxidation reconstruction to provide more exposed active sites (Fig. 2g). In Fig. 2h, HRTEM also detected lattice fringes with a spacing of 0.251 nm, which are attributed to the (− 1 1 1) crystal plane of CuO. Furthermore, High angle annular dark field (HAADF)-STEM-mapping of a single nanorod shows that its elemental composition contains evenly distributed Cu, O and S (Fig. 2i–m). The above characterization results all well prove that the in situ phase transition from Cu_2_S to S-CuO can be successfully achieved without severely damaging the original rod-like structure during this process. A large-scale SEM-mapping by energy-dispersive X-ray spectroscopy (EDS) was used to characterize the elemental composition on the surface of S-CuO/CF samples more accurately. The results show that the doping amount of S in CuO is 5.12% (atomic percentage) (Fig. S6). According to the results of SEM-mapping and TEM-mapping, elements S, Cu and O show uniform distribution of different scales in S-CuO/CF, and their surface and internal element composition are similar (atomic percentage of S is about 5%).

### Performance of Glycerol (Electrochemical) Oxidation

**Fig. 3:**
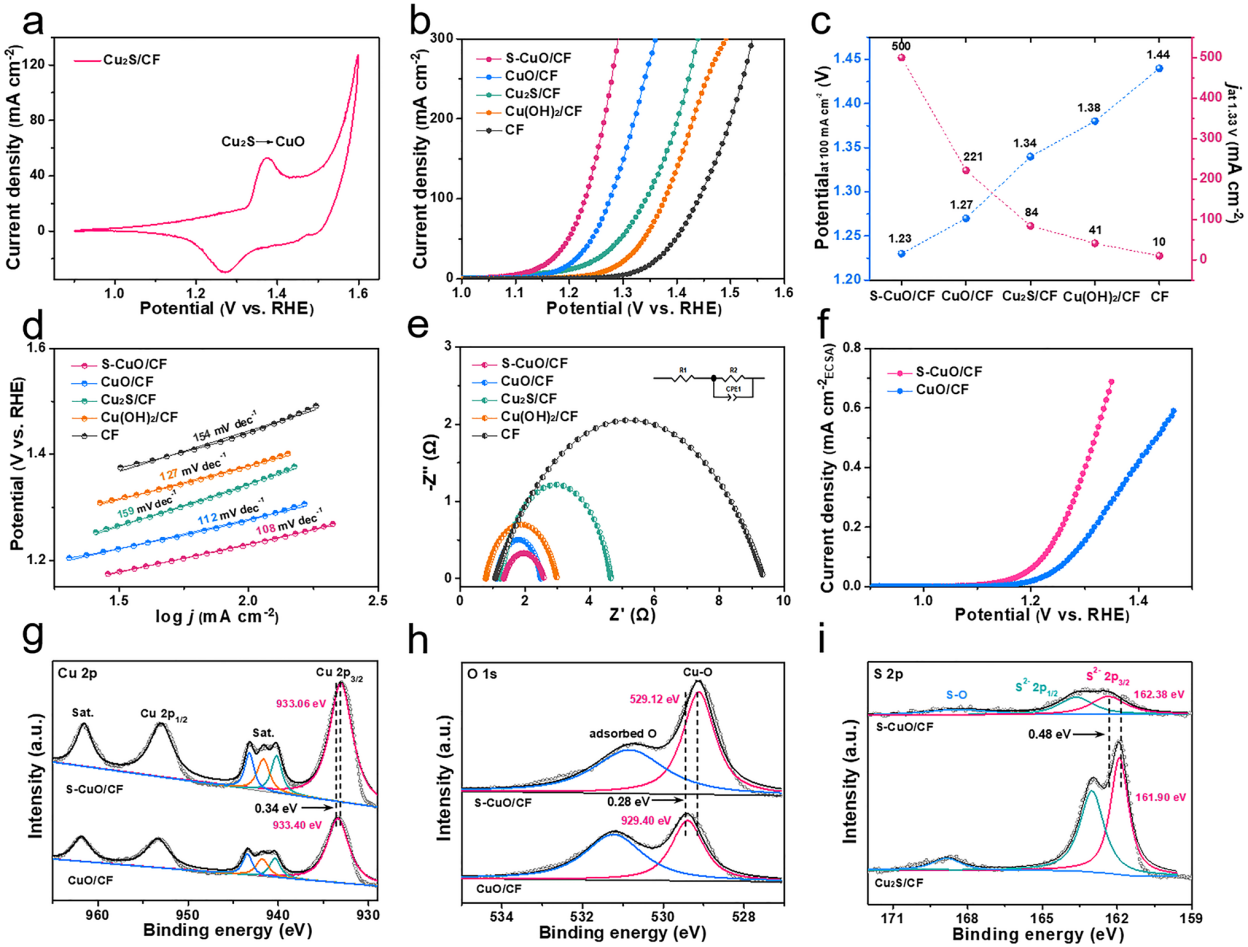
**a** CV test curve of Cu_2_S/CF at 1 M KOH, where oxidation and reduction peaks belong to Cu^+^ to Cu^2+^ transition. **b** GOR polarization curves of S-CuO/CF, CuO/CF, Cu_2_S/CF, Cu(OH)_2_/CF and CF in 1 M KOH solution with 0.1 M glycerol.** c** The potential corresponding to a current density of 100 mA cm^−2^ and the current density generated at 1.33 V versus RHE for all five samples. **d** Tafel slope **e** EIS at 1.25 V versus RHE **f** ECSA-normalized polarization curves of S-CuO/CF and CuO/CF. High-resolution XPS spectra for **g** Cu 2*p* and **h** O 1*s* of S-CuO/CF and CuO/CF. **i** S 2*p* of S-CuO/CF and Cu_2_S/CF

We evaluated the GOR performance of all samples in an alkaline electrolytic cell with typical three electrodes. Figure 3a shows the cyclic voltammetry (CV) scanning curves of Cu_2_S/CF in 1 M KOH, in which there are obvious Cu^+^/Cu^2+^ oxidation and reduction peaks [33, 34]. The Raman spectrum of S-CuO/CF revealed distinct peaks at 280, 334 and 610 cm^−1^ which are associated with CuO reconstructed within an electrochemical environment [33] (Fig. S7). Therefore, the linear sweep voltammetry (LSV) can be operated in the potential range of 1.0–1.75 V versus reversible hydrogen electrode (vs. RHE) to achieve the directional transition of Cu_2_S to S-doped CuO. We extended the in situ electrochemical LSV scanning program from 4 to 20 runs and found that their activation current fluctuated in a small range (Fig. S8a). After 20 scans, there was no significant decrease in GOR properties, indicating that a stable structure could be obtained after the above activation steps (Fig. S8b). Meanwhile, the SEM–EDS mapping of S-CuO(20)/CF shows that the atomic content of S, Cu and O on the material surface is 4.96%, 48.86% and 46.18% (Fig. S9), respectively, which is basically consistent with S-CuO(4)/CF (Fig. S6). In order to highlight the advantages of the structure and properties of S-doped CuO/CF prepared by electroactivation method, CuO/CF was prepared by gas phase oxidation method as a comparison sample. Its XRD diffraction peaks are consistent with the standard cards of CuO(00-001-1117) and three of the strongest peaks were attributed to the metal copper base (Cu 01-070-3038) (Fig. S10). As shown in Fig. S11, the CuO/CF obtained after gas phase oxidation of the precursor still has rod-like structure and rough surface. In 1 M KOH solution with 0.1 M glycerol, LSV curves showed that S-CuO/CF showed significantly enhanced GOR catalytic activity compared with CuO/CF, Cu_2_S/CF, Cu(OH)_2_/CF and CF (Fig. 3b). More specifically, S-CuO/CF only needs 1.23 V to provide current density of 100 mA cm^−2^, which is 40, 110, 150 and 210 mV lower than CuO/CF, Cu_2_S/CF, Cu(OH)_2_/CF and CF, respectively. In addition, S-CuO/CF can reach a high current density of 500 mA cm^−2^ at 1.33 V versus RHE (Fig. 3c), indicating that the introduction of doping S can effectively improve the apparent activity of CuO. In order to further investigate the effect of introducing S element on the intrinsic catalytic activity, we obtained the double layer capacitance (*C*_dl_) of S-CuO/CF and CuO/CF by cyclic voltammetry (CV) (Fig. S12). It is well known that the value of *C*_dl_ is proportional to the electrochemical active surface area (ECSA) of the electrocatalysts. The normalized polarization curve of ECSA showed that the intrinsic catalytic activity of S-CuO/CF is significantly higher than that of CuO/CF (Fig. 3f). Figure 3d shows that S-CuO/CF also exhibits the fastest anodic GOR catalytic reaction kinetics, which can be concluded from its lowest Tafel slope (108 mV dec^–1^). In addition, Electrochemical impedance spectra (EIS) and relevant fitting results indicate that the interface charge transfer resistance of S-CuO/CF (1.242 Ω) is smaller than that of CuO/CF (1.362 Ω), Cu_2_S/CF (3.400 Ω), Cu(OH)_2_/CF (1.899 Ω) and CF (8.296 Ω), because the optimized S-doped CuO surface formed by sulfur leaching and oxidative remodeling is more conducive to interfacial electron transfer and transport (Fig. 3e). More detailed EIS data, including ohmic resistance (*R*_s_) and interfacial charge transfer resistance (*R*_ct_), are included in Table S1.

In order to further reveal the key electronic structure regulation role of doped sulfur in the local microenvironment of CuO, we performed X-ray photoelectron spectroscopy (XPS) on S-CuO/CF and CuO/CF. Figure S13a displays the comprehensive spectrum of Cu(OH)_2_/CF, Cu_2_S/CF, and S-CuO/CF specimens. Correlation peaks for Cu elements were detected in all three specimens, comprising Cu 2*p*, Cu 3*p*, Cu 2*s* and Cu 1*s*. Moreover, the presence of S 2*p* and O 1*s* in the S-CuO/CF confirms the elemental composition of the end product.

Specifically, in the Cu 2*p* spectrum of CuO/CF, peaks located at 933.40 and 953.35 eV are attributed to Cu^2+^ 2*p*_3/2_ and Cu^2+^ 2*p*_1/2_, respectively (Fig. 3g). The incorporation of S causes Cu 2*p*_3/2_ shift 0.34 eV to the lower binding energy, which is because the electronegativity of S is less than that of O, causing partial electron transfer to Cu. The satellite peak of the XPS spectrum consist of three discernible peaks at 940.48, 941.78, and 943.38 eV, respectively. The emergence of these shake-up peaks signifies the Cu holds a valence of + 2 in S-CuO/CF and CuO/CF. This is because the electron configuration of Cu^2+^ is 3*d*_9_, and there are unpaired spintrons in the valence shell, which will be coupled with the inner vacancy, resulting in more than one final state of the system, which is manifested in XPS as spectral line splitting [35–37]. In contrast, in Cu_2_S, Cu has a valence of + 1 and a fully filled 3*d* structure with no vacancy and unpaired electrons, so there are no shake-up peaks (Fig. S13b). Therefore, the Cu 2*p* of Cu_2_S can be divided into Cu^+^ 2*p*_3/2_ (932.80 eV), Cu^+^ 2*p*_1/2_ (952.75 eV) and two satellite peaks, in which the inevitable surface oxidation leads to a small amount of Cu^2+^ (Cu^2+^ 2*p*_3/2_ and Cu^2+^ 2*p*_1/2_). Characteristic peaks of Cu^2+^ 2*p*_3/2_ (934.81 eV) and Cu^2+^ 2*p*_1/2_ (954.76 eV) and complex satellite peaks ascribed to Cu^2+^ have also been found in Cu(OH)_2_/CF, indicating that Cu in it belongs to + 2 valence. Meanwhile, according to the O 1*s* XPS spectra of CuO/CF, the peaks of adsorbed oxygen and lattice oxygen (Cu–O) are positioned at 531.23 and 529.4 eV, respectively (Fig. 3h). The S doping also causes the shift of Cu–O peak to lower binding energies, which indicates that S becomes the main electron deficiency center in the structure and causes the redistribution of CuO electronic structure. In the S 2*p* spectrum of S-CuO/CF, the peaks located at 162.38, 163.68 and 168.48 eV are attributed to S^2−^ 2*p*_3/2_, S^2−^ 2*p*_1/2_ and S–O (arising from adsorbed oxygen from the surface), respectively (Fig. 3i) [38, 39]. When compared with Cu_2_S/CF, the peak of S element in S-CuO/CF demonstrates a significant shift toward higher binding energy, which further substantiates the electron-deficient behavior of doped S. It is noteworthy that the peak strength of S 2*p* in S-CuO/CF has considerably decreased due to the large amount of sulfur leaching that occurred during the electrochemical activation process.

**Fig. 4:**
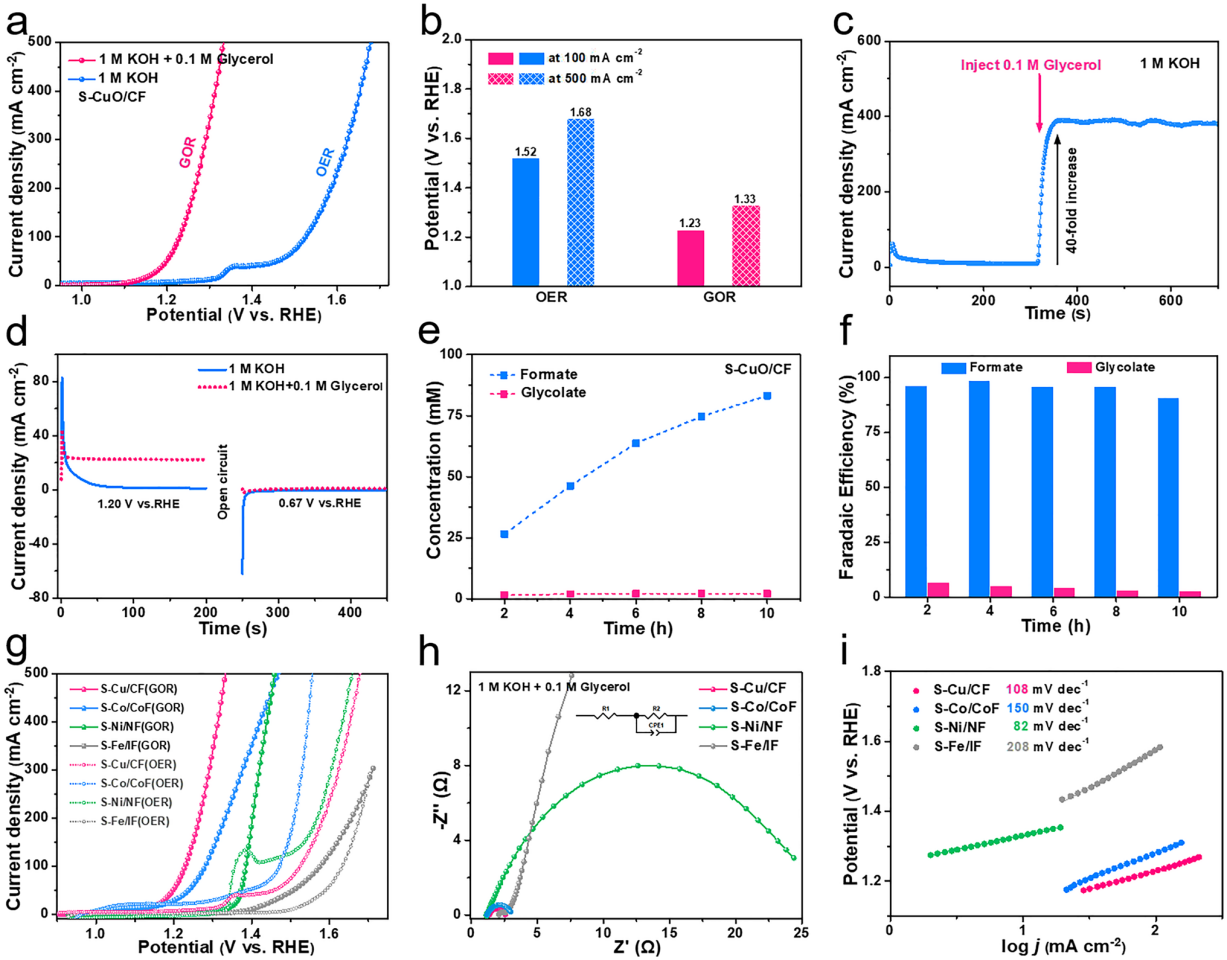
**a** LSV polarization curves and **b** reaction potential (at 100 and 500 mA cm^−2^) of S-CuO/CF in 1 M KOH solution with (GOR) or without (OER) 0.1 M glycerol. **c** The effect of glycerol on the response current. **d** Multi-potential step curves of S-CuO/CF electrode. **e** Concentration changes and **f** Faraday efficiency of the products of S-CuO/CF during the GOR process. **g** LSV polarization curves of S-Cu(O)/CF, S-Co/CoF, S-Ni/NF and S-Fe/IF in 1 M KOH solution with (GOR) or without (OER) 0.1 M glycerol. **h** EIS (at 1.25 V vs. RHE) and **i** Tafel slope of S-Cu(O)/CF, S-Co/CoF, S-Ni/NF and S-Fe/IF in 1 M KOH solution with (GOR) 0.1 M glycerol

In order to further verify the feasibility of GOR as an alternative reaction to OER, the following experiments were conducted. Figure 4a displayed the linear sweep voltammetry (LSV) curves of S-CuO/CF in 1 M KOH with (GOR) and without (OER) 0.1 M glycerol. In the absence of glycerol, the electrode underwent a slow OER. At a current density of 100 mA cm^−2^, the anode potential is 1.52 V versus RHE, and at 500 mA cm^−2^, the potential is as high as 1.68 V versus RHE (Fig. 4b). However, when 0.1 M glycerol was added to the system, the onset potential of the reaction was significantly reduced (1.15 V vs. RHE). And at 100 mA cm^−2^, the anodic GOR reaction potential of is only 1.23 V versus RHE, which is 290 mV lower than OER. At the same time, the Tafel slope of S-CuO/CF for the GOR is much lower than that for the OER, which indicates that this electrocatalyst has faster GOR reaction kinetics (Fig. S14). In addition, compared with other reported transition metal-based GOR electrocatalysts, the S-CuO/CF prepared in this work also showed obvious performance advantages (Table S2) [28, 30, 40–47].

Figure 4c more directly reflects the surprising catalytic performance of S-CuO/CF for GOR. When the system is running at constant potential (1.4 V vs. RHE), the sudden addition of glycerol to the electrolyte results in a 40-fold violent increase in the reaction current, which indicates that S-CuO/CF has a fast and efficient GOR response speed. In addition, in KOH solutions with and without glycerol, S-CuO/CF is held in a higher potential environment (1.20 V vs. RHE) for 200 s, and then the potential was set to the open circuit potential (0.67 V vs. RHE). The results in Fig. 4d show that there is no reduction current in the environment containing glycerol, indicating that the catalytic process of GOR of S-CuO/CF is real and spontaneous.

We used high performance ion chromatography (IC) to collect quantitative reaction product information to reveal the utilization potentiality of glycerol electrooxidation of S-CuO/CF to produce formic acid. During chronoamperometry (CA) (1.35 V vs. RHE) measurement, the composition of the product is analyzed every two hours. The apparent catalytic current in the above process shows a tendency to decay due to the gradual depletion of glycerol (Fig. S15). As shown in Fig. S16, the reaction products always contain only formate and glycolate during the continuous oxidation process. All the products were quantitatively analyzed by standard curve method (Fig. S17). The concentrations of formate and glycolate are counted separately in Fig. 4e. It is worth mentioning that the glycolate concentration was very low and remained basically constant throughout the process, suggesting that it may be an intermediate substance that did not have time to be converted. In addition, as-prepared S-CuO/CF has high Faraday efficiency of 95.7% (average) for catalyzing glycerol to produce formate (Fig. 4f). And with the extension of reaction time, the selectivity of formate production (i.e., relative content of formate) is gradually improved, eventually reaching 95% (Fig. S18). The above results indicate that S-CuO/CF prepared by in situ oxidation reconfiguration method has satisfactory GOR performance.

In order to highlight the generality of the synthesis strategy of S-doped transition metal-based catalytic materials proposed above, we used similar synthesis methods to prepare S-Co/CoF, S-Ni/NF and S-Fe/IF (specific morphology images are given in Fig. S19) on cobalt foam (CoF), nickel foam (NF) and iron foam (IF) and tested their GOR properties respectively. As shown in Fig. 4g, all electrocatalysts showed significantly lower anode potential in the electrolyte containing 0.1 M glycerol. And their GOR catalytic activity follows the following order: S-Cu(O)/CF > S-Co/CoF > S-Ni/NF > S-Fe/IF. Unsurprisingly, S-Cu(O)/CF has the smallest interfacial charge transfer resistance (Table S3), indicating that it has the fastest interfacial electron transfer speed (Fig. 4h). Surprisingly, S-Ni/NF showed the smallest Tafel slope (Fig. 4i). The above abnormal phenomenon can be explained as follows: the intrinsic catalytic activity of S-Ni/NF comes from the formation of Ni^(3+)^OOH during the electrooxidation process. Although S-Ni/NF has the fastest GOR catalytic reaction kinetics, its intrinsic catalytic activity is limited by the difficulty of Ni^(3+)^OOH formation.

**Fig. 5:**
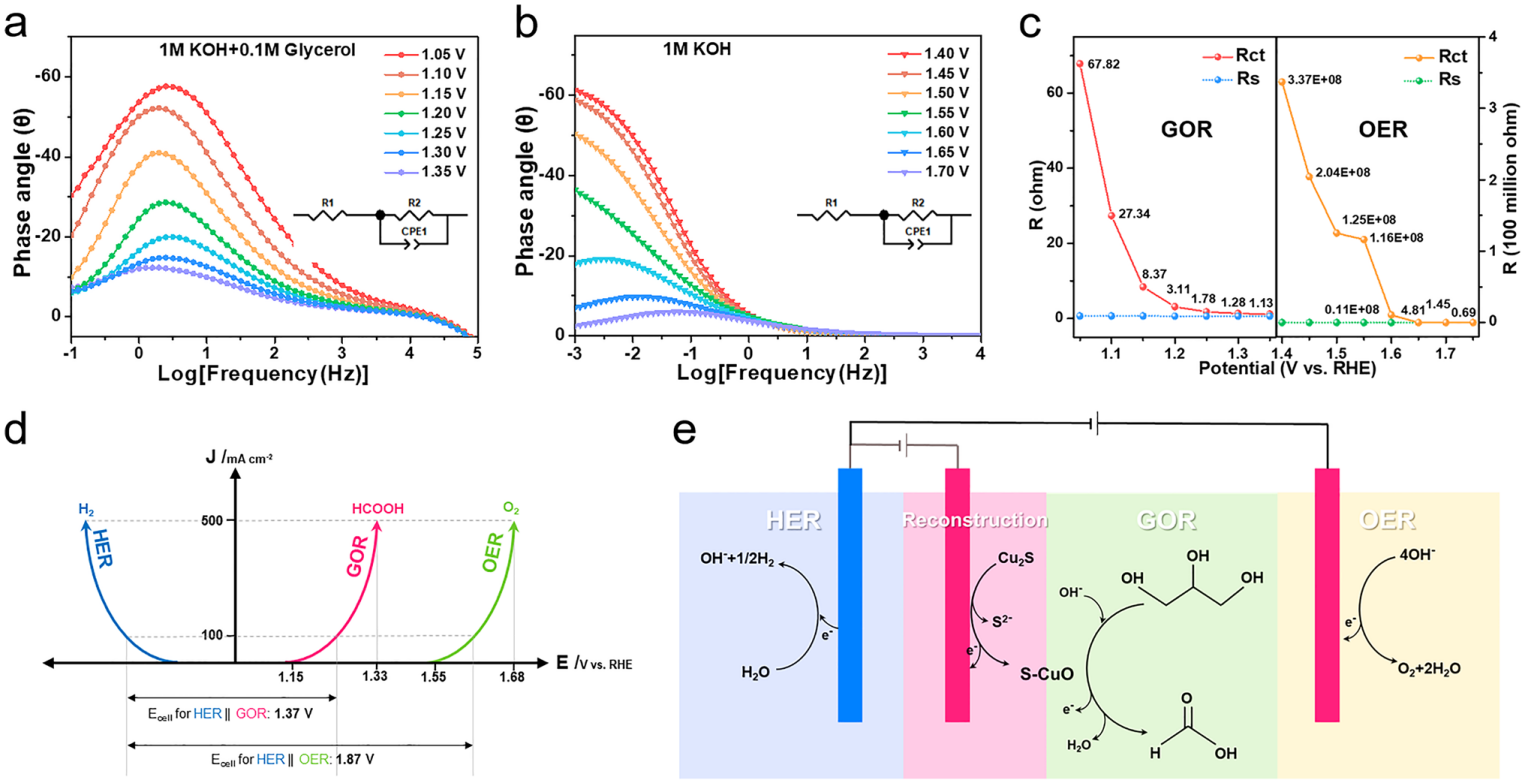
Bode plots of the S-CuO/CF sample in 1 M KOH **a** with and **b** without 0.1 M glycerol. **c** R_ct_ and R_s_ of S-CuO/CF for GOR and OER at different potentials. **d** S-CuO/CF driven GOR and OER processes in relation to potential. **e** Schematic diagram of surface reconstruction of the sample and catalytic reaction processes of GOR and OER

EIS also can be used to compare different interface behaviors related to reaction potential of GOR and OER in detail [48]. The Bode plots of GOR and OER driven by S-CuO/CF are shown in Fig. 5a, b. Among them, it is not difficult to find that that the OER responses are shifted to lower frequency compared to the GOR responses. In the GOR system, a marked reduction in phase angle subsequent to 1.15 V potential signifies the commencement of low-frequency interfacial reaction, which synchronizes with the GOR test current. When the glycerol oxidation potential reaches 1.35 V (the reaction current exceeds 500 mA cm^−2^), the corresponding angular peaks of species oxidation and OER do not appear, which proves that the GOR process is undisturbed, and this is the direct reason for the high Faraday efficiency in this work. For the OER system, when the potential reaches 1.55 V, a weak inflection point appears in the low-frequency region. At this time, the OER interface reaction resistance is 1.16 E + 0.8 Ω and the OER process is really slow. After 1.55 V, *R*_ct_ showed a significant decline, indicating that the OER process unhinderedly occurred between 1.55 and 1.70 V. After fitting the equivalent circuit model (Fig. S20), the R_ct_ and R_s_ of different reaction potentials are counted in Fig. 5c. *R*_ct_ can clearly reflect the interfacial reaction process of the electrocatalyst. Combining the *R*_ct_ and LSV test results, we can draw the following conclusion: (1) S-CuO/CF driven GOR can fully occur in the potential range of 1.15–1.33 V. Thanks to the self-supporting structure covered by the self-derived grown nanorods, S-CuO/CF can provide a high current density of 500 mA cm^−2^ to meet the demand of large-scale industrial generation of hydrogen and formic acid. (2) As depicted in Fig. 5d, OER occurs in the potential range of 1.55 to 1.68 V, completely avoiding competition with GOR, which triggers nearly 100% GOR Faraday efficiency for S-CuO/CF. Based on this, a schematic diagram of the initial catalytic reactions of HER, OER and GOR is presented in Fig. 5e. In conclusion, S-doped CuO nanorods constructed by sulfur leaching and oxidative remodeling performed remarkable electrocatalytic performance of GOR with great potential for industrial application.

**Fig. 6:**
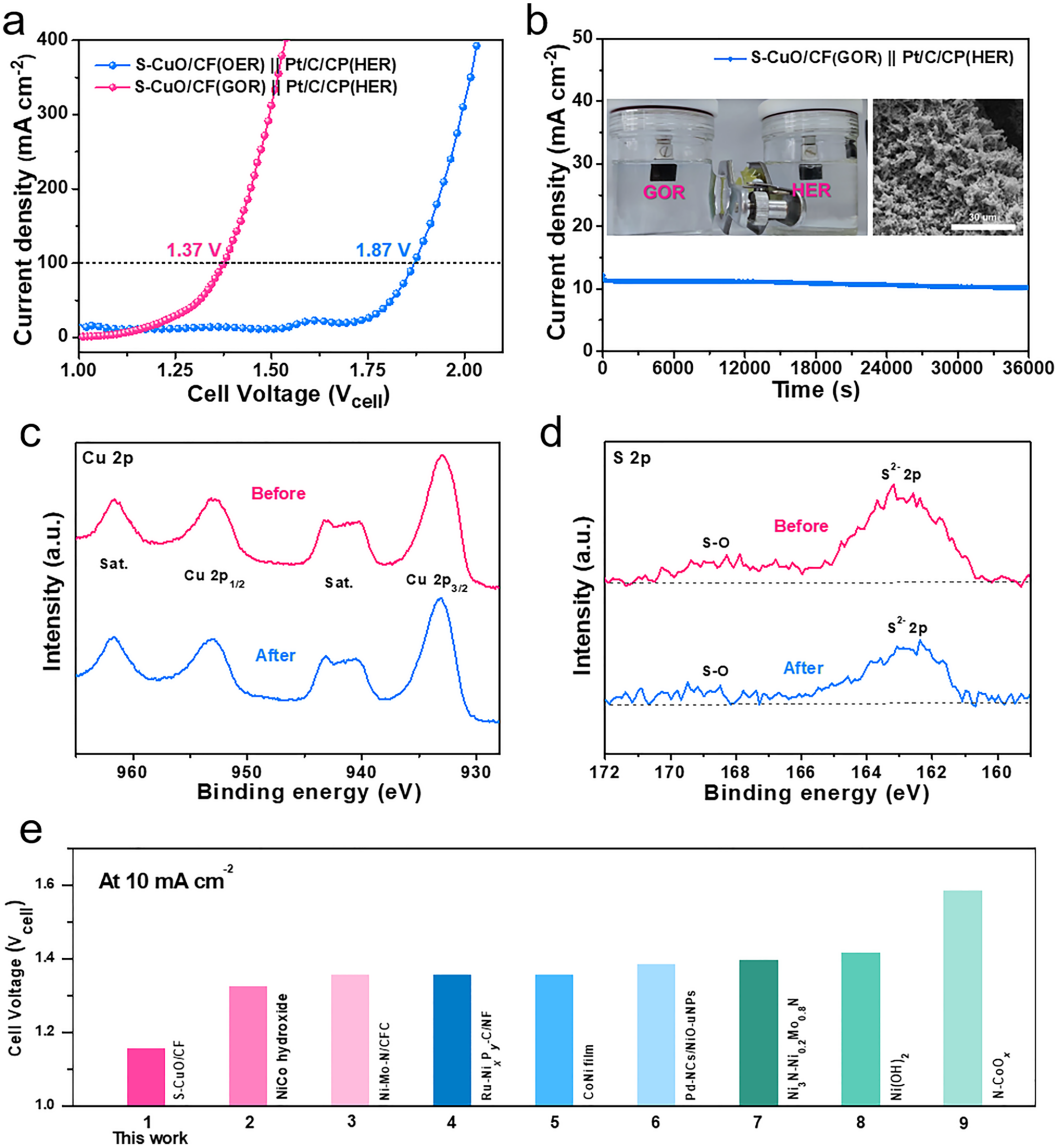
**a** LSV polarization curves for electrolyzer coupling HER (Pt/C/CP) with GOR or OER (S-CuO/CF). **b** Measuring curve of S-CuO/CF(GOR) || Pt/C/CP(HER) electrolytic cell by chronoamperometry (CA) at 1.20 V for 10 h. The illustrations are the pictures of our self-built asymmetric electrolyzer and the surface morphology of the anode electrocatalyst 10 h later. **c** Cu 2*p* and **d** S 2*p* of S-CuO/CF sample before and after stability test. **e** A comparison of the comprehensive properties of organic oxidation coupling HER electrolytic cells driven by other electrocatalysts reported recently

In order to further verify the key application of S-CuO/CF driven GOR as an alternative to OER for anode to reduce energy consumption and improve hydrogen production efficiency, we built an alkaline electrolyzer coupling GOR with HER ((Pt/C/carbon paper (CP)). What makes us excited is that S-CuO/CF(GOR) || Pt/C/CP(HER) cell shows significantly enhanced efficiency. At a current density of 100 mA cm^−2^, it only requires 1.37 V of cell potential (*V*_cell_), which is 500 mV lower than the total water decomposition process (Fig. 6a). In addition, as we can see in Fig. 6e and Table S4 [28, 30, 40–43, 49, 50], the battery pack (S-CuO/CF(GOR) || Pt/C/CP(HER)) reported in this work also has excellent competitiveness for the coupling of organic oxidation and efficient hydrogen production compared with other asymmetric coupling electrolyzers (GOR&HER).Thus, it can not only break through the limitation of slow OER with high energy barrier to greatly reduce the energy consumption of hydrogen production, but it also realizes the efficient preparation of high value-added formic acid. Meanwhile, we also investigated the overall stability of the electrolytic cell assembled by S-CuO/CF electrocatalyst. Based on the results presented in Fig. 6b, after a 10-h stability test, the output current of the electrolytic cell decreased slightly, which may be due to the structural change caused by lattice oxygen deintercalation from the material surface. An XRD analysis of the S-CuO/CF sample conducted following the stability test indicated that the sample composition remained constant throughout the extended testing period (Fig. S21). The XPS spectra portraying the Cu 2*p* of S-CuO/CF revealed analogous peaks before and after the stability examination, thereby indicating the stability of Cu in + 2 valence state (Fig. 6c). As shown in Fig. 6d, the peak corresponding to S^2−^ 2*p* after the stability test displays no considerable shift, but its intensity is estimated to be reduced by about 28% based on peak area. This may be due to some changes in the surface structure of the electrocatalyst during the stability test, resulting in a decrease in the content of S element near the surface. In Fig. S22, the rod-like structure of the electrocatalyst was maintained in general, although some caking occurred. However, we note that the lifetime of this novel S-CuO/CF anode at high current densities needs further improvement in order to inhibit an excessive depletion of lattice oxygen.

### Theoretical Calculation and Catalytic Mechanism

**Fig. 7:**
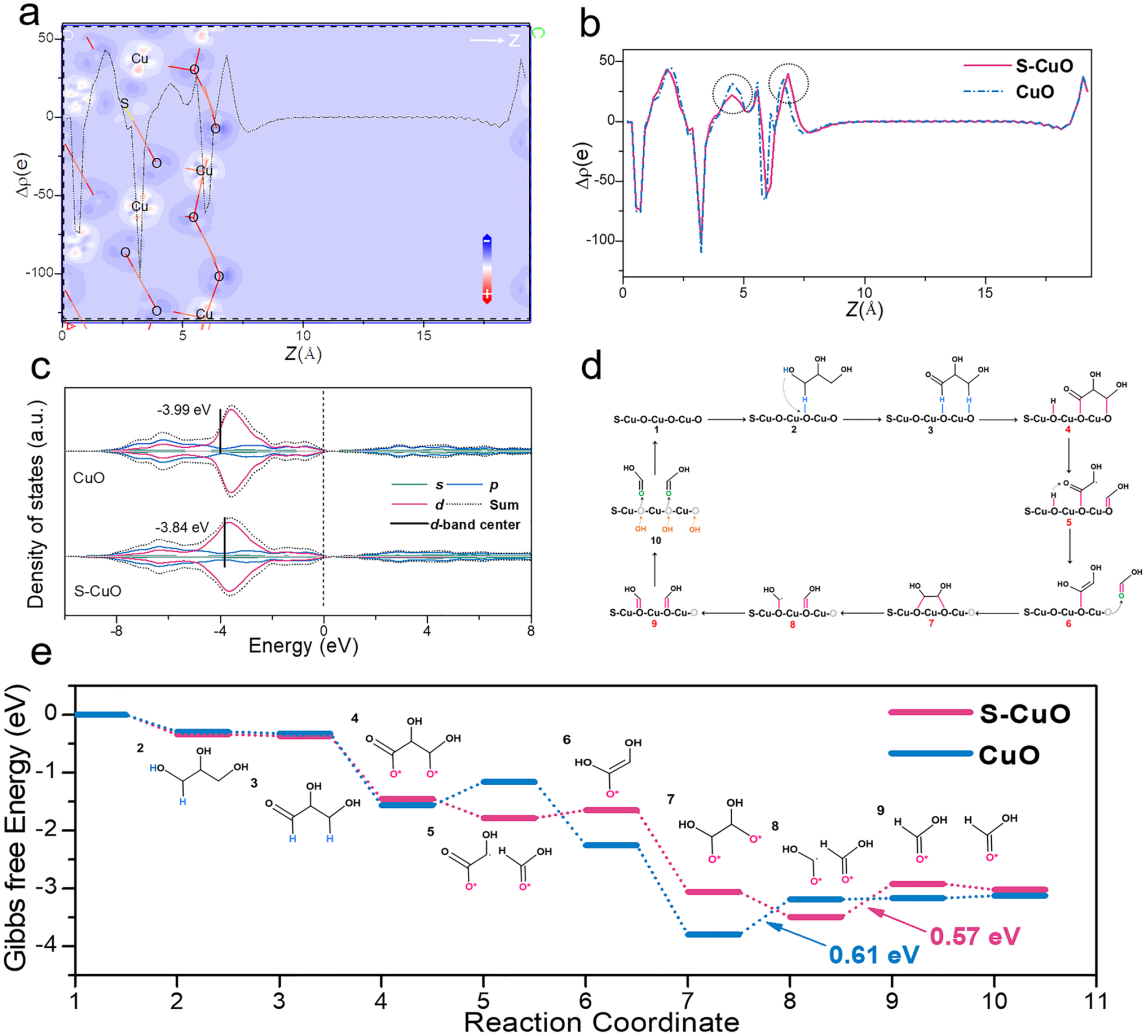
**a** The section of difference charge density of S-CuO. **b** Electron density statistics of S-CuO and CuO along the *Z* axis. **c** Projected density of states (PDOS) of CuO and S-CuO. **d** Cyclic reaction path of S-CuO driven glycerol electrooxidation to prepare formic acid. **e** Calculated Gibbs free energy profiles of GOR on S-CuO and CuO. The numerical data are shown in Table S5

Combined with the above experimental results and related reports, we performed the DFT calculation to reveal the detailed reaction pathway and regulatory mechanism of glycerol electrooxidation. In order to better analyze the electron interaction between different atoms, we calculated the difference charge density of CuO and S-CuO respectively (Fig. S23). The section of charge density of S-Cuo is shown in Fig. 7a. Figure 7b illustrates the electron density statistics of CuO (− 1 1 1) and S-CuO (− 1 1 1) across the vacuum layer direction. Upon S-doping, a reduction in electron concentration is observed in the bulk phase layer accompanied by electron accumulation in the surface layer (visible in the circles of Fig. 7b) when compared to CuO. This may be more conducive to catalyzing the interaction between the surface and reactant molecules. Further, the Gibbs free energy diagrams of GOR on CuO and S-CuO are summarized in Fig. 7e. For both CuO and S-CuO, the initial dehydrogenation steps (1–2, 2–3 and 3–4) are spontaneous, suggesting that they are ideal surfaces for glycerol adsorption and catalytic oxidation.

Subsequent C–C bond breaking (4–5 and 7–8) [44, 51], intermediate isomerization (5–6 and 8–9) and lattice oxygen deintercalation (5–6, 9–10 and 7′-8′) processes play key role in the whole catalytic cycle [28]. For CuO, the first C–C bond breaking step (4–5) is spontaneous, while the second C–C bond breaking (7–8) becomes the rate-determining step (RDS) for the entire formic acid formation, with a maximum energy change of 0.61 eV. For S-CuO, both C–C bond breaking steps during the oxidation of glycerol to formic acid are spontaneous. This is because S doping causes more electrons to transfer to the O atoms on the catalytic surface, which is more conducive to the electron interaction between the intermediates and catalytic surface to induce active bond-breaking process. However, this strong electron interaction makes the subsequent intermediate isomerization and lattice oxygen deintercalation steps (5–6 and 8–9) more difficult to be spontaneous. Thus, for S-CuO, the second intermediate isomerization (8–9) with an energy requirement of 0.57 eV becomes the RDS of the entire process, which determines the catalytic performance of oxidized glycerol for formic acid production. By comparing the calculated energy difference between steps 5–6 and 9–10 of the catalytic reaction, we can know that the selective oxidation capacity of the electrocatalysts is determined by the lattice oxygen deintercalation process [28]. Too strong oxygen anion deintercalation capacity will result in the formation of different intermediate products and poor formic acid production selectivity. On the contrary, if the oxygen anion deintercalation ability is too weak, the whole catalytic process will be blocked. In this work, S-doping-induced interfacial electron redistribution gives S-CuO the most moderate lattice oxygen deintercalation capacity, which results in a high production selectivity for formic acid up to 95%. In Fig. 7c, the PDOS of CuO and S-CuO indicated that the d-band center of the material shifted upward due to S doping, which was more conducive to strengthening the electronic coupling between the catalytic surface and glycerol oxidation intermediates to promote the GOR process. On this basis, we propose a reasonable cyclic catalytic path for S-CuO induced GOR process (Figs. 7d and S24).

## Conclusions

In summary, we prepared S-CuO nanorods array structure using sulfur leaching and oxidative remodeling strategy to catalyze the production of formate by GOR and couple it with HER for efficient hydrogen generation. S-CuO/CF showed satisfactory GOR catalytic activity (1.23 V vs. RHE at 100 mA cm^−2^) and driving ability of asymmetric coupling electrolyzer (1.37 V_cell_ at 100 mA cm^−2^). Detailed electrochemical data, physical characterization and mechanism analysis results indicate that excellent catalytic performance of S-CuO/CF results from fully exposed active sites, rapid interfacial charge transfer and optimized electronic structure. Meanwhile, the moderate lattice oxygen deintercalation ability of it leads to high formic acid production selectivity. This work fully demonstrates the feasibility of the designed transition metal oxides as low energy consumption electrocatalysts for the oxidation of glycerol to formate and coupled hydrogen evolution.

### Supplementary Information

Below is the link to the electronic supplementary material.

Supplementary file1 (PDF 1964 KB)
