# Supplementary material for: Optimized Electronic Modification of S-Doped CuO Induced by Oxidative Reconstruction for Coupling Glycerol Electrooxidation with Hydrogen Evolution
Source: Nanomicro Lett. 2023 Jul 29;15:190. doi: 10.1007/s40820-023-01159-6 (PMC10386980; doi:10.1007/s40820-023-01159-6)
Supplement: Supplementary file 1 — Supplementary file1 (PDF 1964 KB) [file 40820_2023_1159_MOESM1_ESM.pdf]

Nano-Micro Letters

Supporting Information for

Optimized Electronic Modification of S-Doped CuO Induced by Oxidative Reconstruction for Coupling Glycerol Electrooxidation with Hydrogen Evolution

Ruo-Yao Fan[1], Xue-Jun Zhai[1], Wei-Zhen Qiao[1], Yu-Sheng Zhang[1], Ning Yu[1], Na Xu[1], Qian-Xi Lv[1], Yong-Ming Chai[1,]*, Bin Dong[1,]*

[1] China State Key Laboratory of Heavy Oil Processing, College of Chemistry and Chemical Engineering, China University of Petroleum (East China), Qingdao 266580, P. R. China

*Corresponding authors. E-mail: ymchai@upc.edu.cn (Yong-Ming Chai); dongbin@upc.edu.cn (Bin Dong)

S1 Experimental Section

S1.1 Synthesis of CuO/CF

CuO/CF was obtained by calcining $Cu(OH)_2$/CF in air at 350°C for 1 hour.

S1.2 Synthesis of S-Co/CoF, S-Ni/NF and S-Fe/IF

Copper foam (CoF), nickel foam (NF) and iron foam (IF) are purchased from Kunshan Lvchuang Electronic Technology Co., LTD directly. Their thickness and porosity specifications are as follows: CoF 1.5 mm, 110 ppi; NF 1.5 mm, 110 ppi; IF 1.5 mm, 95 ppi. The CoF and NF are subjected to ultrasonic cleaning using acetone, hydrochloric acid and ethanol, respectively, prior to their utilization. The iron mesh, on the other hand, underwent ultrasonic cleaning solely with acetone and ethanol.

Firstly, $Co(OH)_2$/CoF, $Ni(OH)_2$/NF and $Fe(OH)_3$/IF precursors were prepared by traditional hydrothermal method. Taking $Co(OH)_2$/CoF as an example, the specific method was to dissolve 2 mmol $Co(NO_3)_2$•$6H_2O$ and 10 mmol urea in 30 mL deionized water and pour it into a 100 mL hydrothermal crystallization reactor. A piece of CoF (10*20*1.5 mm) was dipped into the solution and leaned vertically against the inner wall. After the assembled reactor was kept at 100°C for 10 hours, the $Co(OH)_2$/CoF was prepared. The preparations of $Ni(OH)_2$/NF and $Fe(OH)_3$/IF were similar to the above process, except that $Co(NO_3)_2$•$6H_2O$ was replaced by $Ni(NO_3)_2$•$6H_2O$ and $Fe(NO_3)_3$•$9H_2O$, and CoF was replaced by NF and IF, respectively. Then, the obtained $Co(OH)_2$/CoF, $Ni(OH)_2$/NF and $Fe(OH)_3$/IF were treated with hydrothermal vulcanization, a procedure consistent with the preparation of S-CuO/CF.

S1.3 Preparation of Pt/C Cathode

We utilized a commercial Pt/C cathode (with a mass loading of 3 mg cm^{-2}) that was

coated onto carbon paper (CP) (10*10*0.3 mm, HCP030N). The formulation for the coating consists of 0.03 g of Pt/C, 1 g of isopropyl alcohol and 0.5 g of deionized water. It is crucial to ensure that the slurry is effectively dispersed via ultrasonic means before application via spraying.

S1.4 Materials Characterization

The crystal phase of the obtained catalysts was studied via X-ray diffraction (XRD) on a Rigaku D/max-2500pc device with Cu Kα radiation (λ = 1.54 Å). The scanning electron microscope (SEM) (Hitachi S-4800) and Transmission Electron Microscope (TEM) (FEI Tecni G20, 200 kV) were applied to collect the information of morphological and structural information of all samples. The element composition and distribution on catalysts were detected by means of the Energy Dispersive System (EDS) and detected on the Hitachi S-4800. The X-ray photoelectron spectroscopy (XPS) is characterized by a Thermo Fisher Scientific II spectrometer with an Al Kα source (1486.6 eV).

S1.5 Electrochemical Measurements

The catalytic performance of all the catalysts was evaluated in a standard three-electrode cell configuration on Gamry Reference 1000 electrochemical equipment at room temperature. This process was performed with the Pt foil (10*10 mm), Hg/HgO electrode and catalyst samples (10*10 mm) as the counter electrode, reference electrode, and working electrode, respectively. The OER and GOR performance tests utilized electrolytes of 1 M KOH and 1 M KOH + 0.1 M glycerol, respectively. All 1 M KOH used in this work are pre-saturated with N_2. The LSV were gained at same condition with scan rate of 5 mV s^{-1} with iR correction (Current interruption (CI) compensation). All the potentials vs. Hg/HgO were converted into a standard reversible hydrogen electrode (RHE) by means of the Nernst equation: $E_{RHE} = E_{Hg/HgO} + 0.0594\ pH + 0.098$. The cyclic voltammetry (CV) curves were tested at 40, 60, 80, 100, 120 mV s^{-1} and were used to determine the electrical double-layer capacitances (C_{dl}). The specific calculation equation is as follows:

$$C_{dl} = (ja-jc)/(2\bullet v) = (ja+|jc|)/(2\bullet v) = \Delta j/(2\bullet v) \qquad \text{(S1)}$$

in which ja and jc is the anodic and cathodic voltammetric current density, respectively, recorded at the middle of the selected potential range, and v is the scan rate.

The electrochemcial active surface area (ECSA) in this work is calculated by the following publicity:

$$ECSA = C_{dl}/C_{dl,\ ideal} \qquad \text{(S2)}$$

in which $C_{dl,\ ideal}$ is the double layer capacitance of an ideally flat electrode, which is commonly taken as 40 μF cm^{-2} in alkaline media.

Electrochemical impedance spectroscopy (EIS) was performed at 1.25 V vs. RHE and the frequency ranges from 10^5 Hz to 0.1 Hz. The stability of the final sample was performed by chronoamperometry (CA).

S1.6 Product Analysis

The oxidation products of glycerol were determined by ion chromatography (IC) with as9-hc column and conductivity detector. A long-term electrolytic reaction of glycerol oxidation was performed at a constant potential of 1.35 V vs. RHE in 200 mL electrolyte of 1 M KOH and 0.1 M glycerol. 0.5 mL electrolyte was extracted every 2 hours and diluted 10 times with deionized water as the solution to be tested. 0.954 g sodium carbonate was dissolved in 1000 mL secondary water as eluent at a constant flow rate of 0.6 mL/min.

The corresponding faradaic efficiencies (FE) toward formate and glycolate are calculated based on the following equations:

$$FE_{formate} = \frac{C_{formate} \times \frac{1}{3} \times 8}{Q_{total}} \times V \times F \times 100\% \quad (S3)$$

$$FE_{glycolate} = \frac{C_{glycolate} \times \frac{2}{3} \times 5}{Q_{total}} \times V \times F \times 100\% \quad (S4)$$

where $C_{formate}$ and $C_{glycolate}$ are the final concentrations of formate and glycolate, respectively; V is the volume of the electrolyte solution; F is the Faraday's constant (96485 C/mol); The integration of the j-t response curve (chronoamperometry) yields the Q_{total}, representing the total charge transfer.

The Faradaic efficiency calculations of the glycerol oxidation production are based on the following balance half-reactions:

$$C_3H_8O_3\ (glycerol) + \frac{13}{2}\ OH^- \rightarrow \frac{3}{2}\ C_2H_3O_3^-\ (glycolate) + 5\ H_2O + 5\ e^- \quad (S5)$$

$$C_3H_8O_3\ (glycerol) + 11\ OH^- \rightarrow 3\ HCOO^-\ (formate) + 8\ H_2O + 8\ e^- \quad (S6)$$

S2 Computational Methods

The density functional theory (DFT) calculations were carried out by CASTEP. The exchange-correlation interaction between atomic cores and valence electrons with DFT was described by generalized gradient approximation (GGA) with the Perdew-Burke-Ernzerhof (PBE) functional. Plane-wave cutoff energy of 500 eV was used in all computations. The total energy and force convergence threshold was set as $2*10^{-5}$ eV/atom and 0.05 eV/Å. A three-layer slab with a p(3×2) supercell was used to simulate the CuO (-111) and S-CuO (-111) surfaces, and the bottom layer was fixed to mimic bulk properties. For surfaces geometry optimization, a Monkhorst-Pack k-point mesh of 2×2×1 was employed. The d-band centers were determined by taking the weighted mean energy of the projected density of states (pDOS) of metal 3d states relative to the Fermi level.

The Gibbs free energy (ΔG) of glycerol oxidation on CuO (-111) and S-CuO (-111) was calculated by correcting the obtained total energy with zero-point energy and

entropy. The calculation of Gibbs free energy was modeled using computational equation (S7):

$$\Delta G=\Delta E+\Delta ZPE-T\Delta S \qquad (S7)$$

where ΔE is the total energy difference, ΔZPE is the difference of zero-point energy, ΔS is the change of entropy, and T is the environment temperature (298 K).

S3 Supplementary Figures

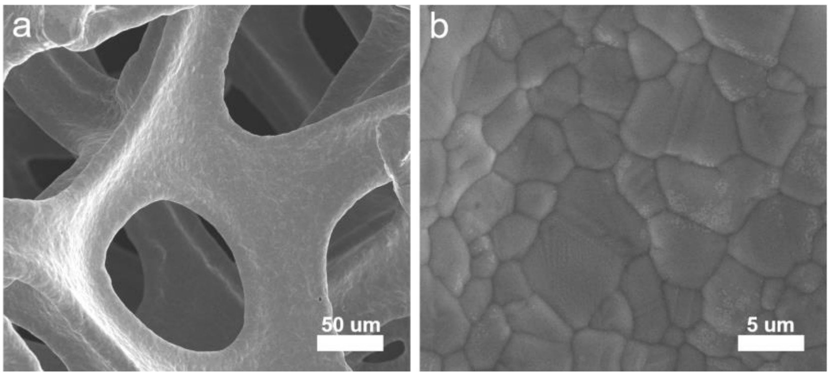

Fig. S1 SEM images of CF

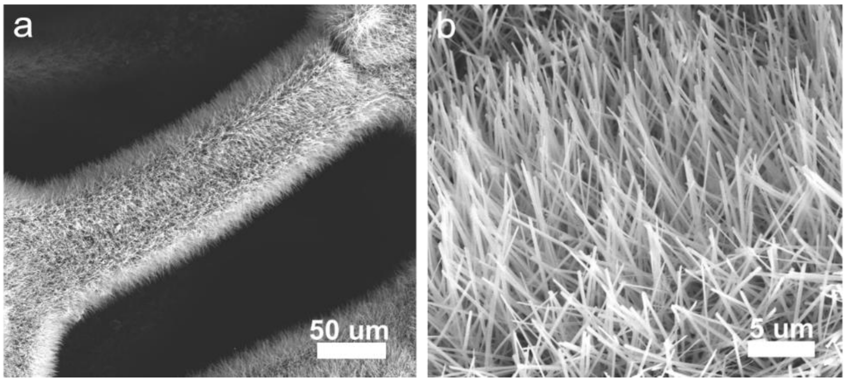

Fig. S2 SEM images of $Cu(OH)_2$/CF

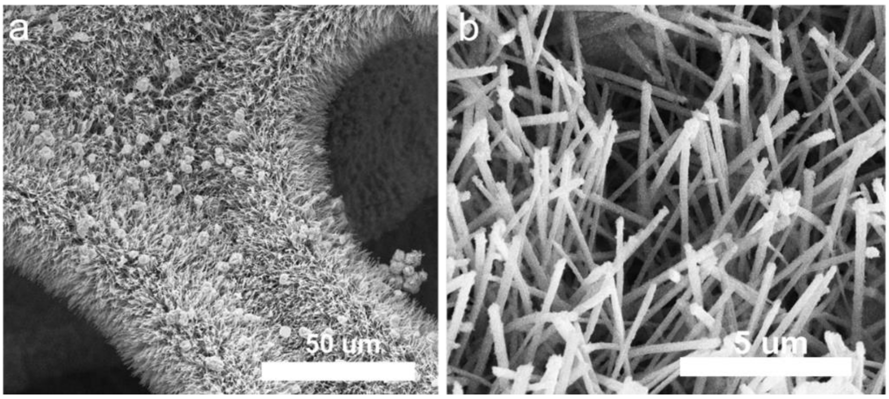

Fig. S3 SEM images of Cu_2S/CF

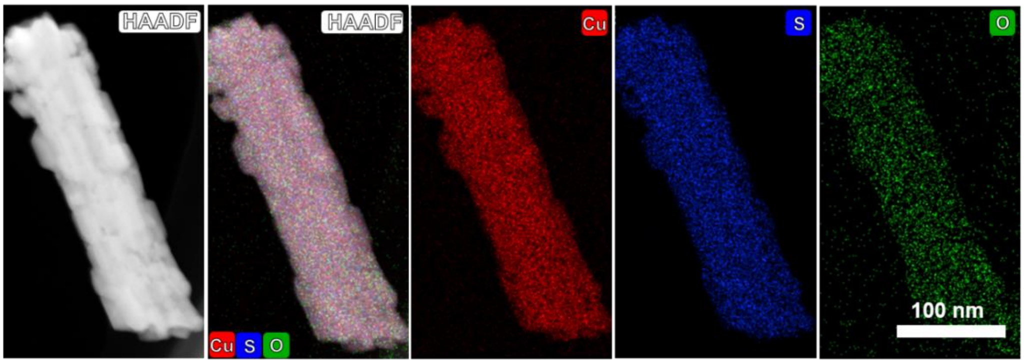

Element	Linellae	Atomic Fraction (%)	Atomic Error (%)	Mass Fraction (%)	Mass Error (%)	Fit error (%)
O	K	4.33	0.84	1. 30	0.15	2.23
S	K	26.69	7.14	16.12	3.52	0.13
Cu	K	68.98	16.13	82.57	14.48	0.15
Total		100.00		100.00		

Fig. S4 TEM-EDS-mapping of Cu_2S/CF (with the oxygen distribution)

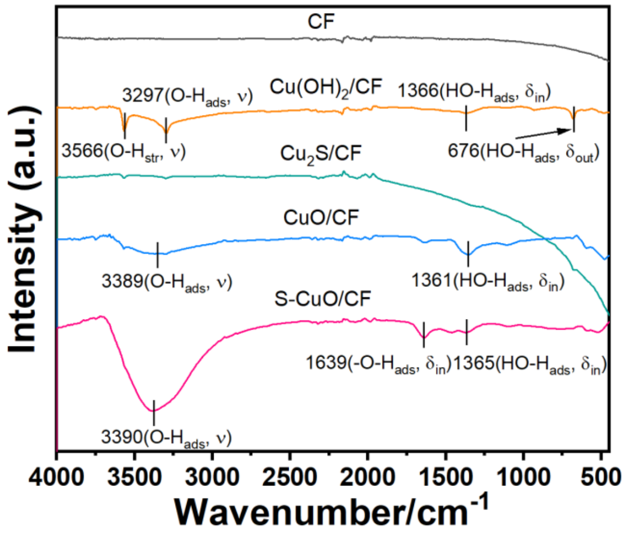

Fig. S5 FTIR spectra of bare CF, $Cu(OH)_2$/CF , Cu_2S/CF, CuO/CF and S-CuO/CF

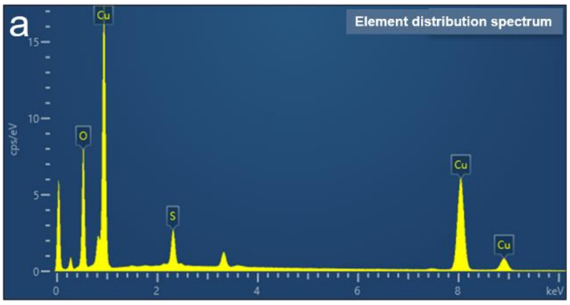

Element	Linellae	apparent concentration	k ratio	Wt%	At%	standard sample
O	K	36.02	0.12120	22.61	52.35	SiO_2
S	K	4.60	0.03963	4.44	5.12	FeS_2
Cu	K	88.02	0.88016	72.95	42.53	Cu
Total				100.00	100.00	

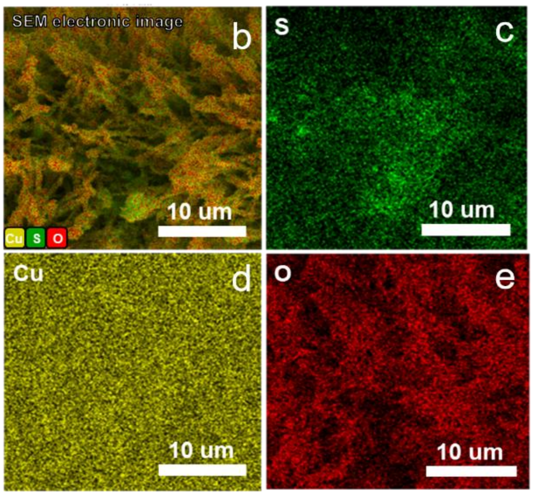

Fig. S6 The SEM-EDS-Mapping of S-CuO/CF

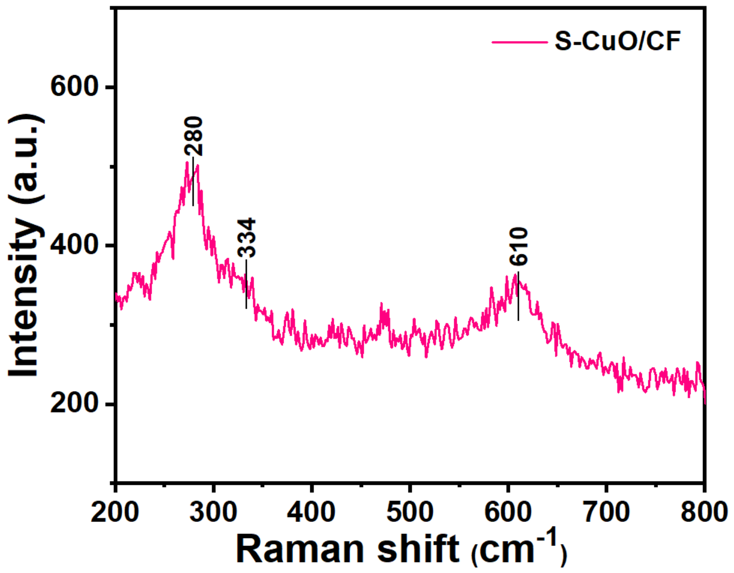

Fig. S7 Raman spectrum of S-CuO/CF

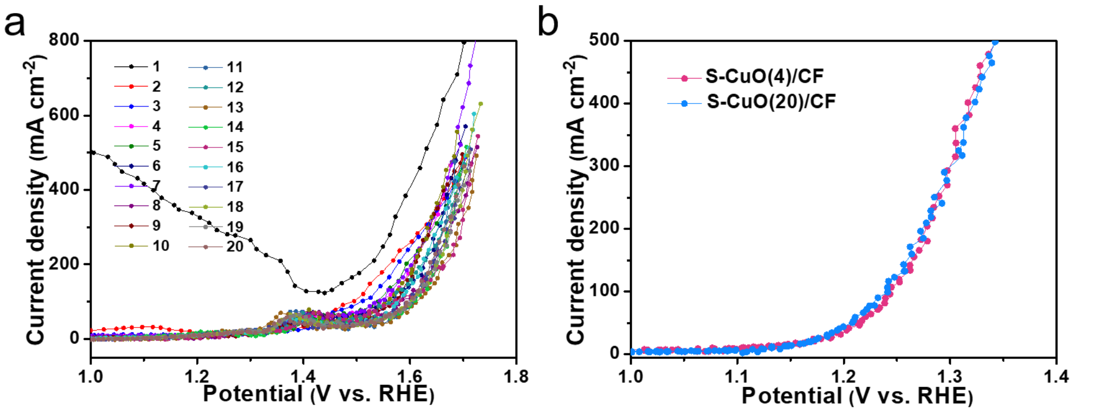

Fig. S8 (**a**) In 1 M KOH, 20 times in situ electrochemical LSV activation curves. (**b**) GOR test curves of S-CuO/CF activated by 4 (S-CuO(4)/CF) and 20 times (S-CuO(20)/CF) LSV in 1 M KOH with 0.1 M glycerol, respectively

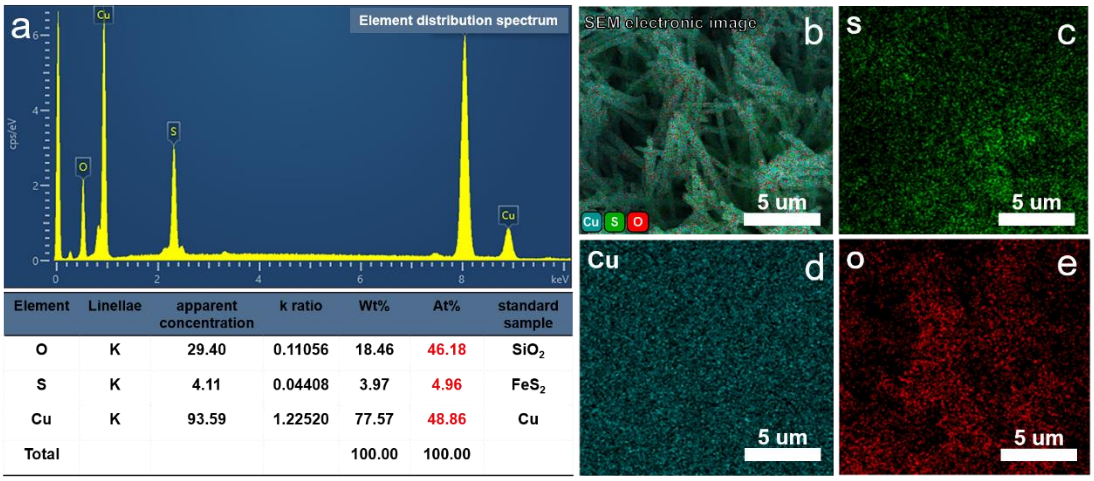

Element	Linellae	apparent concentration	k ratio	Wt%	At%	standard sample
O	K	29.40	0.11056	18.46	46.18	SiO_2
S	K	4.11	0.04408	3.97	4.96	FeS_2
Cu	K	93.59	1.22520	77.57	48.86	Cu
Total				100.00	100.00	

Fig. S9 SEM-EDS-mapping of S-CuO(20)/CF

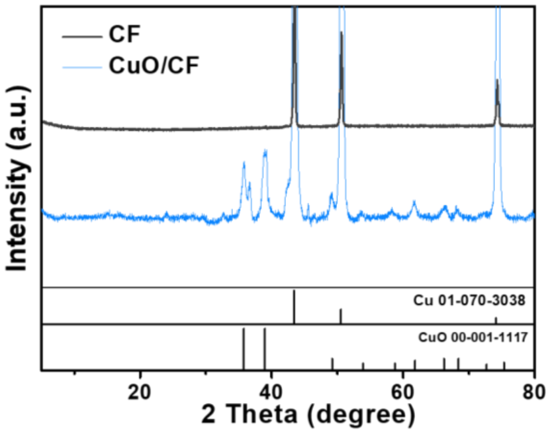

Fig. S10 XRD of CF and CuO/CF

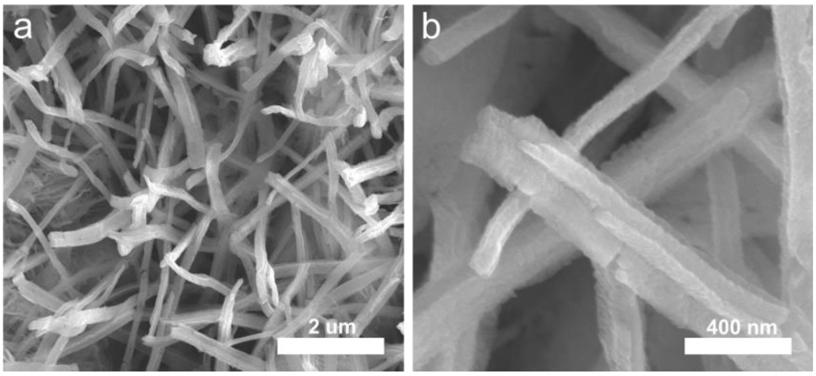

Fig. S11 The SEM of CuO/CF

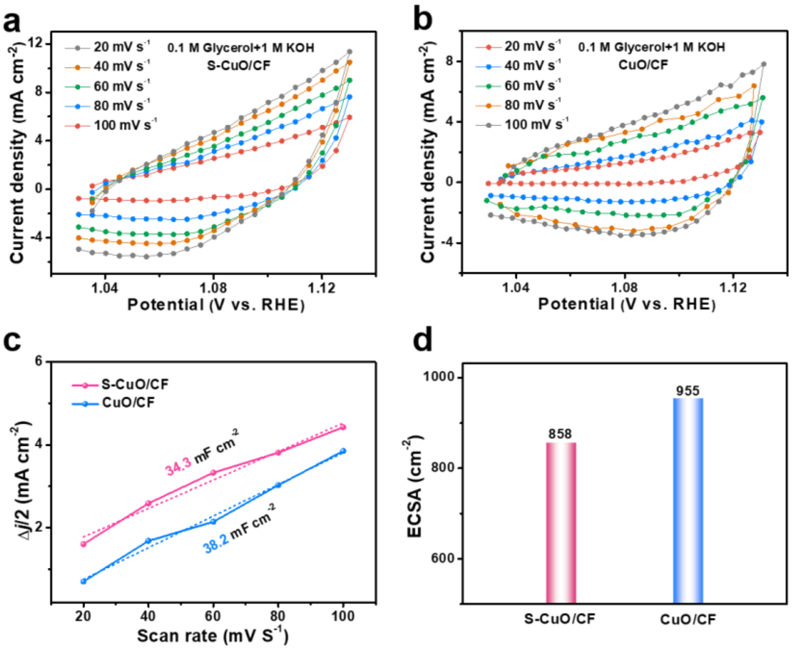

Fig. S12 The CV of (**a**) S-CuO/CF and (**b**) CuO/CF. (**c**) C_{dl} and (d) ECSA of S-CuO/CF and CuO/CF

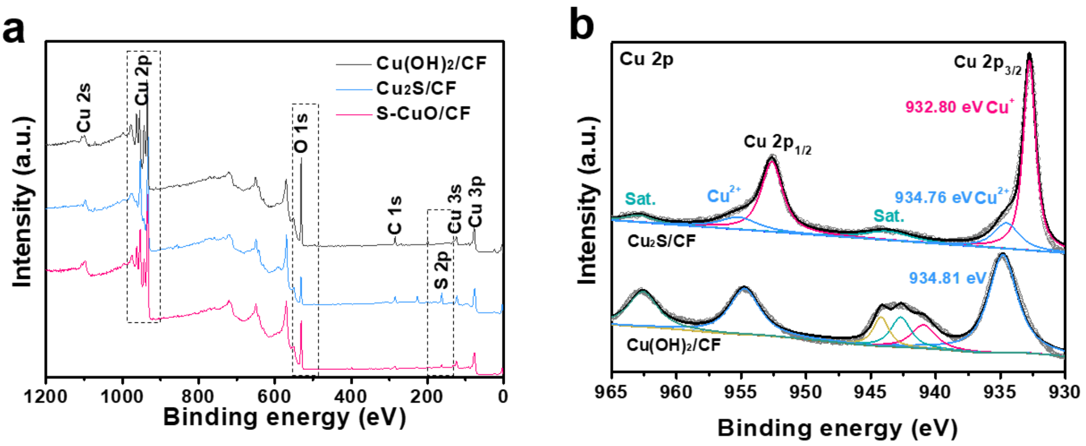

Fig. S13 (**a**) The survey XPS of $Cu(OH)_2$/CF, Cu_2S/CF and S-CuO/CF. (**b**) The Cu 2*p* of Cu_2S/CF and $Cu(OH)_2$/CF

Table S1 R_s and R_{ct} of S-CuO/CF, CuO/CF and Cu_2S/CF

	R_s/Ω	R_{ct}/Ω
S-CuO/CF	1.342	1.242
CuO/CF	1.121	1.362
Cu_2S/CF	1.246	3.400
$Cu(OH)_2$/CF	0.7848	1.899
CF	1.071	8.296

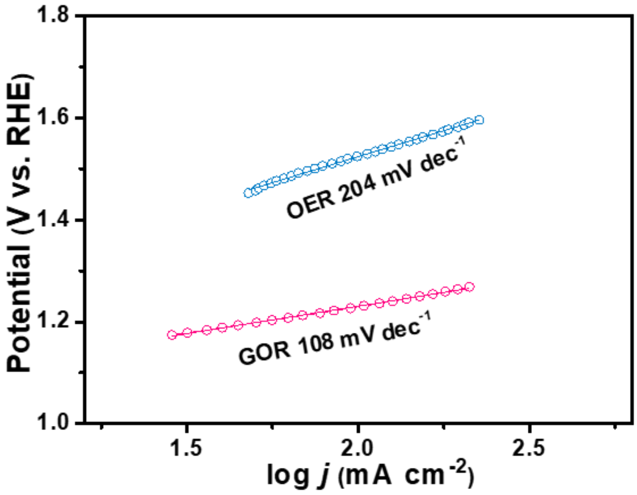

Fig. S14 The Tafel slope of S-CuO/CF for OER and GOR

Table S2 Comparison of GOR properties of S-CuO/CF measured in a three-electrode system with other recently reported transition metal based electrocatalysts

Electrocatalysts	Solution composition	Potential at 10 mA cm^{-2}	Potential at 100 mA cm^{-2}	Tafel slope (mV dec^{-1})	References
S-CuO/CF	0.1 M Glycerol+1 M KOH	1.13 V	1.23 V	108	This work
NiCo hydroxide	0.1 M Glycerol+1 M KOH	-	1.35 V	68.8	[28]
Ni-Mo-N/CFC	0.1 M Glycerol+1 M KOH	1.30 V	1.51 V	87	[30]
Ru-Ni$_x$P$_y$/N-C/NF	0.1 M Glycerol+1 M KOH	1.32 V	1.4 V	86	[41]
CoNi film	0.33 M Glycerol+1 M KOH	1.30 V	1.44 V	124	[42]
Pd-NCs/NiO-uNPs	0.50 M Glycerol+1 M KOH	-	1.43 V	57.5	[43]
Ni_3N-$Ni_{0.2}Mo_{0.8}N$ NWs/CC	0.1 M Glycerol+1 M KOH	1.30 V	1.41 V	96	[44]
N-CoO$_x$	1 M Glycerol+1 M KOH	1.31 V	-	155	[46]
CuCo-oxide	0.1 M Glycerol+0.1 M KOH	1.25 V	-	90±2.757	[47]
Ni_3N/Co_3N-NWs	0.1 M Glycerol+1 M KOH	1.18 V (20)	1.26V (50)	131.4	[49]
Cu-Cu_2O/CC	0.5 M Glycerol+1 M KOH	1.21 V	1.4 V	111.7	[50]
Cu-CuS/BM	0.1 M Glycerol+0.1 M KOH	1.37 V	-	-	[51]

(The numbers in parentheses represent current density, such as 1.18 V (20) represents the potential at 20 mA cm^{-2}.)

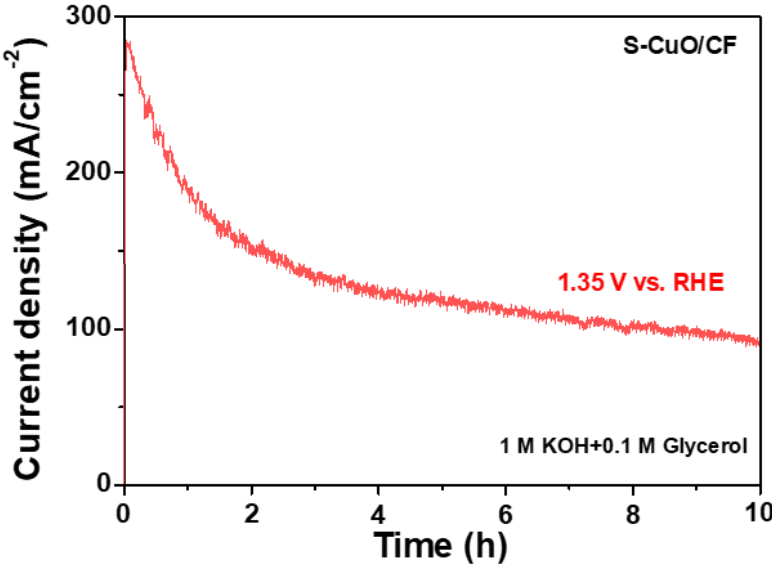

Fig. S15 CA test curve of S-CuO/CF at 1.35 V vs. RHE in 200 mL electrolyte of 1 M KOH with 0.1 M glycerol

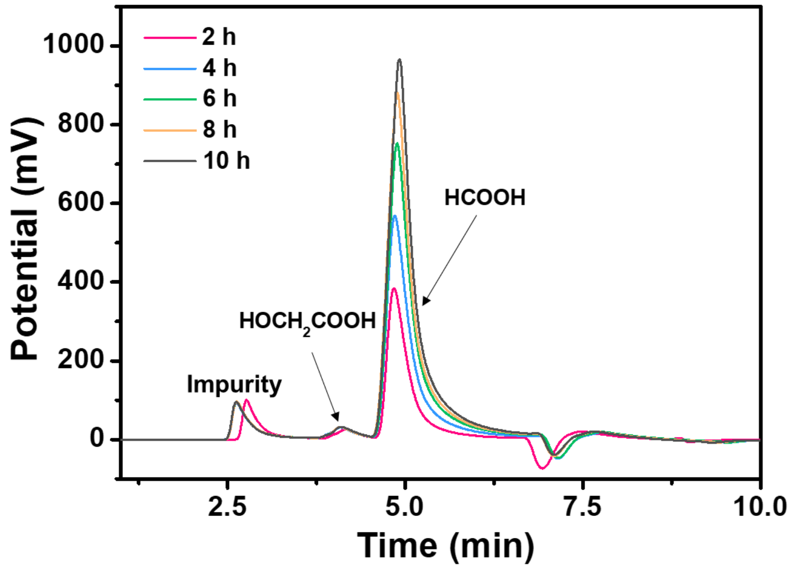

Fig. S16 The evolution of IC chromatograms as a function of electrolyzing time. The impurity peaks may be caused by the trace amount of F^- contained in the ultra-pure water. Its content does not change during the whole process and there is no interference to the analysis of the product. The electrolysis experiments were carried out on a constant applied potential of 1.35 V vs. RHE in 200 mL electrolyte of 1 M KOH with 0.1 M glycerol.

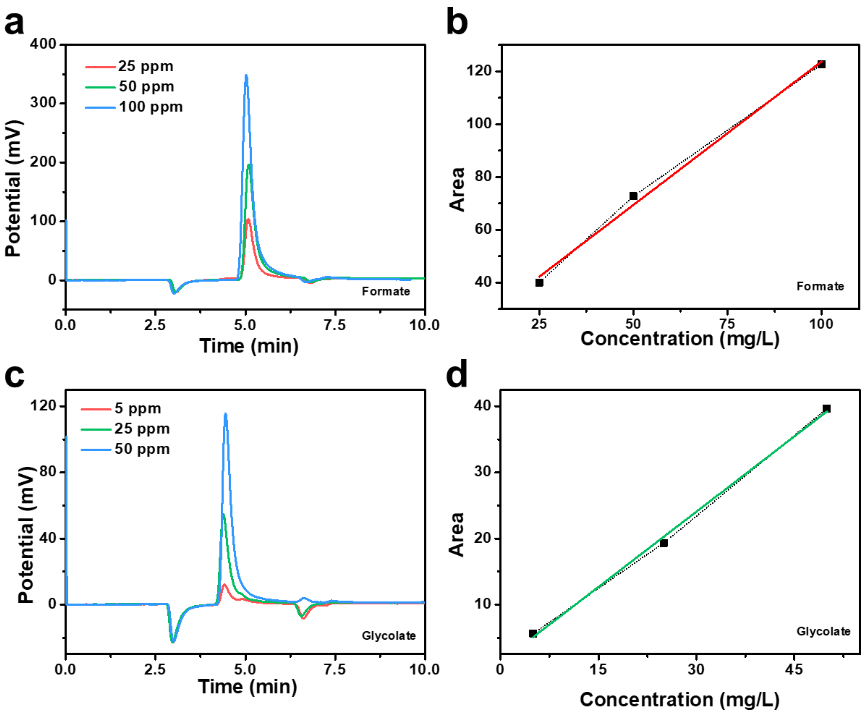

Fig. S17 Standard high-performance ion chromatography (IC) chromatograms and the corresponding calibration curves of ((a) and (b)) formate and ((c) and (d)) glycolate

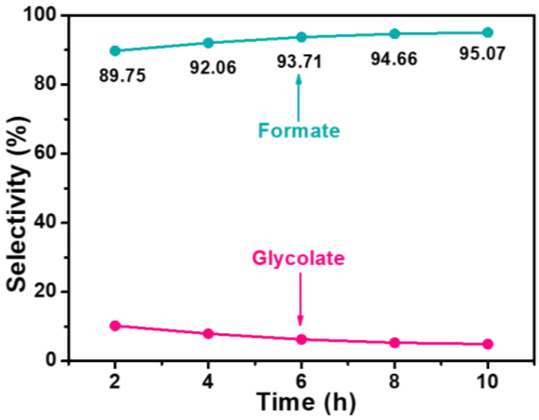

Fig. S18 The selectivity of formate and glycolate over time in a continuous GOR process catalyzed by S-CuO/CF

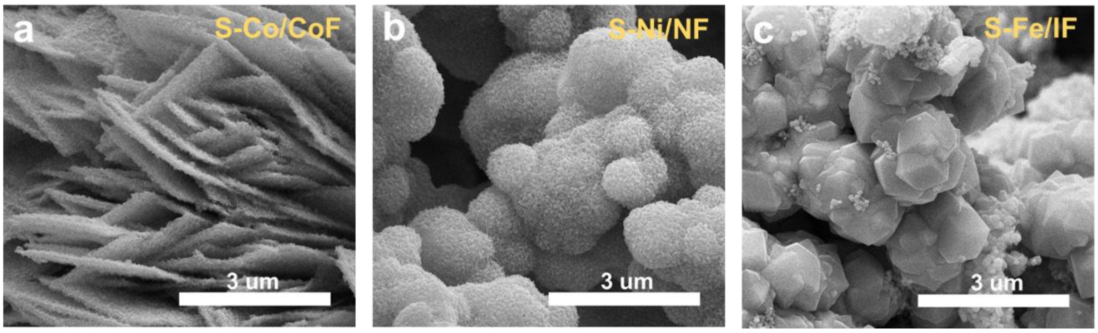

Fig. S19 SEM images of (**a**) S-Co/CoF, (**b**) S-Ni/NF and (**c**) S-Fe/IF

Table S3 R_s and R_{ct} of S-Cu(O)/CF,S-Co/CoF, S-Ni/NF and S-Fe-IF

	R_s/Ω	R_{ct}/Ω
S-Cu/CF	1.342	1.242
S-Co/CoF	1.183	1.908
S-Ni/NF	1.164	25.48
S-Fe/IF	2.608	∞

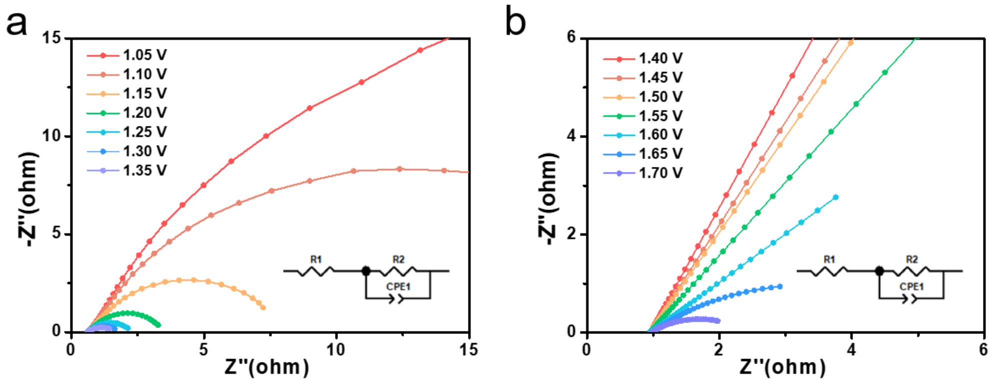

Fig. S20 Nyquist curves of S-CuO/CF in (a) 1 M KOH with 0.1 M glycerol and (b) 1 M KOH

Table S4 A comparative analysis of other reports on asymmetric coupling electrolyzers intended for glycerol oxidation coupled with efficient hydrogen production

**Anode (GOR) catalysts**	**Cathode (HER) catalysts**	**Solution composition**	**V_{cell} at 10 mA/cm^{-2}**	**V_{cell} at 100 mA/cm^{-2}**	**Reference**
S-CuO/CF	Pt/C/CP	0.1 M Glycerol+1 M KOH	1.16 V	1.37 V	This work
NiCo hydroxide	NiCo hydroxide	0.1 M Glycerol+1 M KOH	1.33 V	1.58 V	[28]
Ni-Mo-N/CFC	Ni-Mo-N/CFC	0.1 M Glycerol+1 M KOH	1.36 V	-	[30]
Ru-Ni*x*P*y*/N–C/NF	Ru-Ni*x*P*y*/N–C/NF	0.1 M Glycerol+1 M KOH	1.36 V	-	[41]
CoNi film	CoNi film	0.33 M Glycerol+1 M KOH	1.36 V	-	[42]
Pd-NCs/NiO-uNPs	Pd-NCs/NiO-uNPs	0.50 M Glycerol+1 M KOH	1.62 V	-	[43]

Ni_3N-$Ni_{0.2}Mo_{0.8}N$ NWs/CC	Ni_3N-$Ni_{0.2}Mo_{0.8}N$ NWs/CC	0.1 M Glycerol+1 M KOH	1.40 V		[44]
$Ni(OH)_2$	$NiCrO_x$/Ni	0.1 M Glycerol+2 M LiOH	1.42 V		[45]
N-CoOx	N-CoOx	1 M Glycerol+1 M KOH	1.59 V		[46]
Ni_3N/Co_3N-NWs	Ni_3N/Co_3N-NWs	0.1 M Glycerol+1 M KOH	1.47 V (50)	1.59 V	[49]

(The numbers in parentheses represent current density, such as 1.47 V (50) represents the cell potential at 50 mA cm^{-2}.)

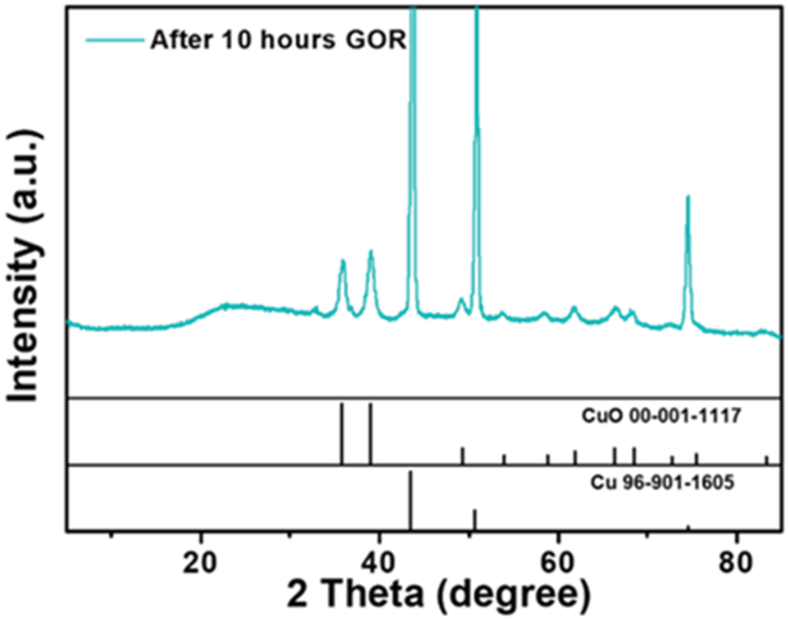

Fig. S21 XRD of S-CuO/CF after stability test

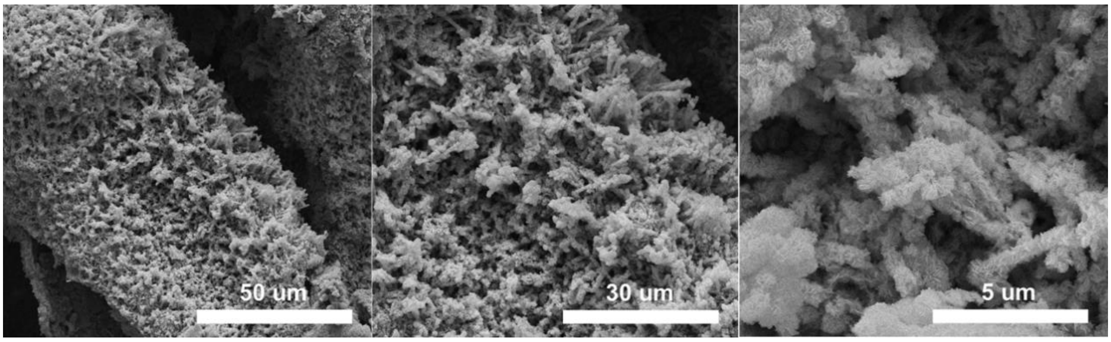

Fig. S22 SEM of S-CuO/CF after stability test

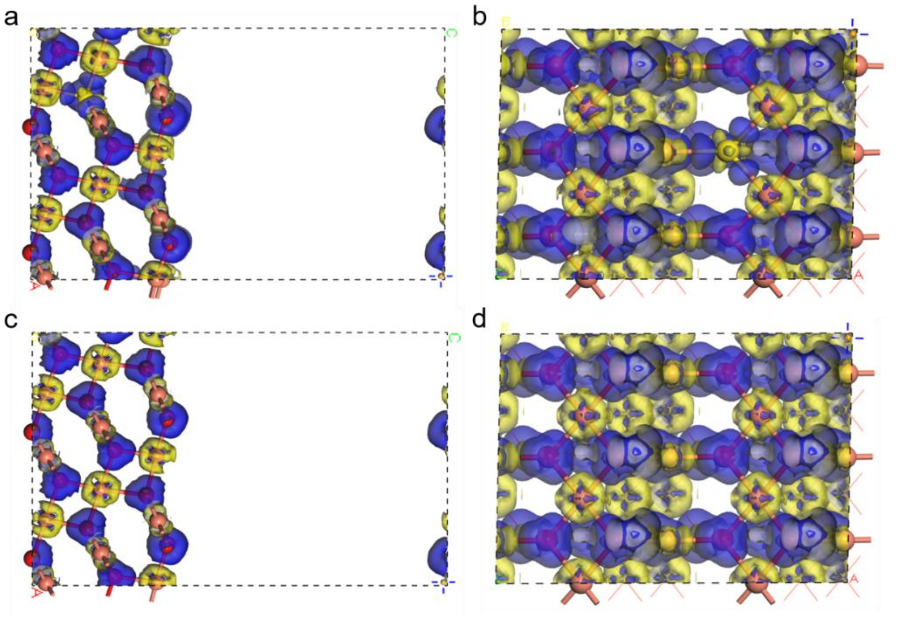

Fig. S23 The difference charge density side view and top view of S-CuO (a and b) and CuO (c and d). The O, Cu and S atoms are represented by the red, orange and yellow spheres, respectively. The blue and yellow translucent outlines represent electron accumulation and loss, respectively

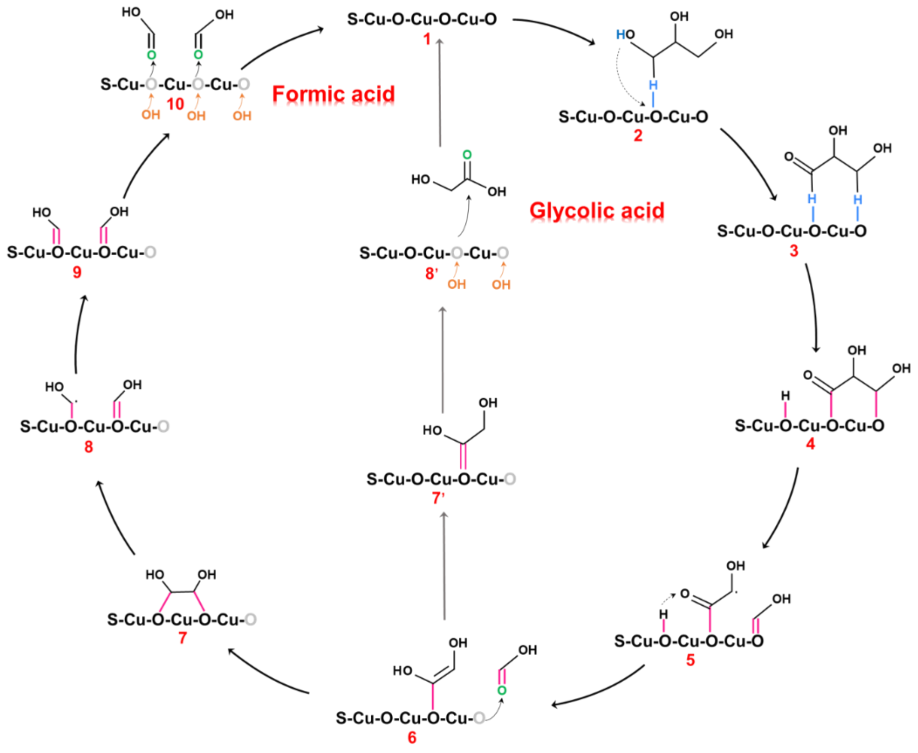

$$S\ CuO\ +\ C_3H_8O_3 \rightarrow S\ CuO\ +\ C_3H_8O_3 \qquad (1\text{-}2)$$

$$S\ CuO\ +\ C_3H_8O_3 \rightarrow S\ CuO\ +\ C_3H_6O_3\ + 2H\cdot \qquad (2\text{-}3)$$

$$S\ CuO\ +\ C_3H_6O_3\ \rightarrow S\ CuO(H)\ +\ C_3H_4O_3\ + H\cdot \qquad (3\text{-}4)$$

$$S\ CuO(H) + C_3H_4O_3 + H \cdot\rightarrow S\ CuO + C_2H_3O_2 + CH_2O \qquad (4\text{-}5)$$

$$S\ CuO + C_2H_3O_2 + CH_2O \rightarrow S\ CuO^* + C_2H_3O_2 + CH_2O_2 \qquad (5\text{-}6)$$

$$S\ CuO^* + C_2H_3O_2 + H \cdot\rightarrow S\ CuO^* + C_2H_4O_2 \qquad (6\text{-}7)$$

$$S\ CuO^* + C_2H_4O_2 \rightarrow S\ CuO^* + CH_2O \cdot + CH_2O \qquad (7\text{-}8)$$

$$S\ CuO^* + CH_2O \cdot + CH_2O \rightarrow S\ CuO^* + CH_2O + CH_2O \qquad (9\text{-}10)$$

$$S\ CuO^* + CH_2O + CH_2O \rightarrow S\ CuO^{3*} + CH_2O_2 + CH_2O_2 \qquad (10\text{-})$$

(* stands for oxygen vacancy)

Fig. S24 The cyclic reaction path of S-CuO/CF driven glycerol electrooxidation to prepare formic acid and glycolic acid

Table S5 Gibbs free energy of each step in GOR

Step	S-CuO/eV	CuO/eV
1-2	-0.34133	-0.28996
2-3	-0.02157	-0.03293
3-4	-1.08642	-1.23994
4-5	-0.33619	+0.40687
5-6	+0.13929	-1.09938
6-7	-1.41273	-1.54194
7-8	-0.43474	+0.60956
8-9	+0.57358	+0.02101
9-10	-0.10002	+0.04220
